# Inherited resilience to clonal hematopoiesis by modifying stem cell RNA regulation

**DOI:** 10.1126/science.adx4174

**Published:** 2026-01-01

**Authors:** Gaurav Agarwal, Mateusz Antoszewski, Xueqin Xie, Yash Pershad, Uma P. Arora, Chi-Lam Poon, Peng Lyu, Andrew J. Lee, Chun-Jie Guo, Tianyi Ye, Laila Barakat Norford, Anna-Lena Neehus, Lucrezia della Volpe, Lara Wahlster, Diyanath Ranasinghe, Tzu-Chieh Ho, Trevor S. Barlowe, Arthur Chow, Alexandra Schurer, James Taggart, Benjamin H. Durham, Omar Abdel-Wahab, Kathy L. McGraw, James M. Allan, Ruslan Soldatov, Alexander G. Bick, Michael G. Kharas, Vijay G. Sankaran

**Affiliations:** 1 Division of Hematology/Oncology, Boston Children’s Hospital, Harvard Medical School, Boston, MA, USA.; 2 Department of Pediatric Oncology, Dana-Farber Cancer Institute, Harvard Medical School, Boston, MA, USA.; 3 Howard Hughes Medical Institute, Boston Children’s Hospital, Boston, MA, USA.; 4 Broad Institute of MIT and Harvard, Cambridge, MA, USA.; 5 Molecular Pharmacology Program, Memorial Sloan Kettering Cancer Center, New York, NY, USA.; 6 Division of Genetic Medicine, Department of Medicine, Vanderbilt University Medical Center, Nashville, TN, USA.; 7 Computational Oncology Service, Department of Epidemiology and Biostatistics, Memorial Sloan Kettering Cancer Center, New York, NY, USA.; 8 PhD Program in Biomedical Informatics, Harvard Medical School, Boston, MA, USA.; 9 Translational and Clinical Research Institute, Newcastle University Centre for Cancer, Faculty of Medical Sciences, Newcastle University, Newcastle upon Tyne, UK.; 10 Immune Deficiency Cellular Therapy Program, National Cancer Institute, National Institutes of Health, Bethesda, MD, USA.; 11 Laboratory of Receptor Biology and Gene Expression, National Cancer Institute, National Institutes of Health, Bethesda, MD, USA.; 12 Myeloid Malignancies Program, National Institutes of Health, Bethesda, MD, USA.; 13 Harvard Stem Cell Institute, Cambridge, MA, USA.

## Abstract

Somatic mutations that increase the fitness of hematopoietic stem cells (HSCs) drive their expansion in clonal hematopoiesis (CH) and predispose individuals to blood cancers. Population variation in the growth rate and potential of mutant clones suggests that genetic factors may confer resilience against CH. Here, we identified a noncoding regulatory variant, rs17834140-T, that protects against CH and myeloid malignancies by selectively down-regulating the RNA-binding protein MSI2 in HSCs. By modeling variant effects and mapping MSI2 binding targets, we uncovered an RNA network that maintains human HSCs and influences CH risk. Variant rs17834140-T was associated with slower CH expansion, and stem cell MSI2 levels modified *ASXL1*-mutant HSC clonal dominance. These findings leverage natural resilience to illuminate posttranscriptional regulation in human HSCs, suggesting that inhibition of MSI2 or its targets could be rational strategies for blood cancer prevention.

Healthy aging tissues harbor a substantial burden of cancer driver mutations, underscoring the widespread nature of somatic mosaicism (1). In the hematopoietic system, myeloid malignancy (MyM)–associated mutations are commonly detected in healthy individuals, a phenomenon termed clonal hematopoiesis (CH). Some mutations in hematopoietic stem and progenitor cells (HSPCs) confer a fitness advantage (2), causing pervasive positive selection and expansion with age (3, 4). Individuals with detectable CH of indeterminate potential (CHIP) above ~2% in peripheral blood have an elevated lifetime risk of developing MyMs (5) and a range of chronic disorders (6-8), including cardiovascular disease (6, 9). However, although there has been marked progress in describing the natural history and consequences of CH, the mechanisms that drive the dominance of stem cell clones remain poorly understood.

Recently, mechanistic investigations based on genome-wide association studies (GWASs) (10-14) have identified genetic regulators of stem cell clonality. For example, functional follow-up of inherited MyM risk variants has revealed that increased HSC self-renewal drives MyM predisposition (11, 14-17). By contrast, the germline mechanisms that protect HSCs from CH and MyMs remain poorly understood. Although CH driver mutations occur ubiquitously in aging adults (18), most never progress to a large clone size or overt MyM. Moreover, lineage tracing has demonstrated that driver mutations may be acquired decades before disease, suggesting that there are mechanisms that restrain expansion (19, 20). Indeed, longitudinal profiling (3, 21-23) and inference of clonal expansion rates (17) show that the growth rate and potential of mutant HSC clones vary greatly. Although chronic inflammation (24) and chemical exposures (25) could contribute to this variable expansion, these observations suggest that germline genetic variation might also promote resilience to CH and MyMs.

Here, we leveraged human genetic variation to identify an inherited mechanism that protects HSCs from CHIP, through down-regulation of the RNA-binding protein MSI2. By modeling variant effects in primary human hematopoietic cells and conducting mechanistic studies, we uncovered an MSI2-regulated RNA network that maintains human HSCs and that is attenuated through genetic variation to resist stem cell expansion and progression to MyMs.

## Identification of a CHIP and MyM resilience haplotype at the 17q22 locus

**Fig. 1.**
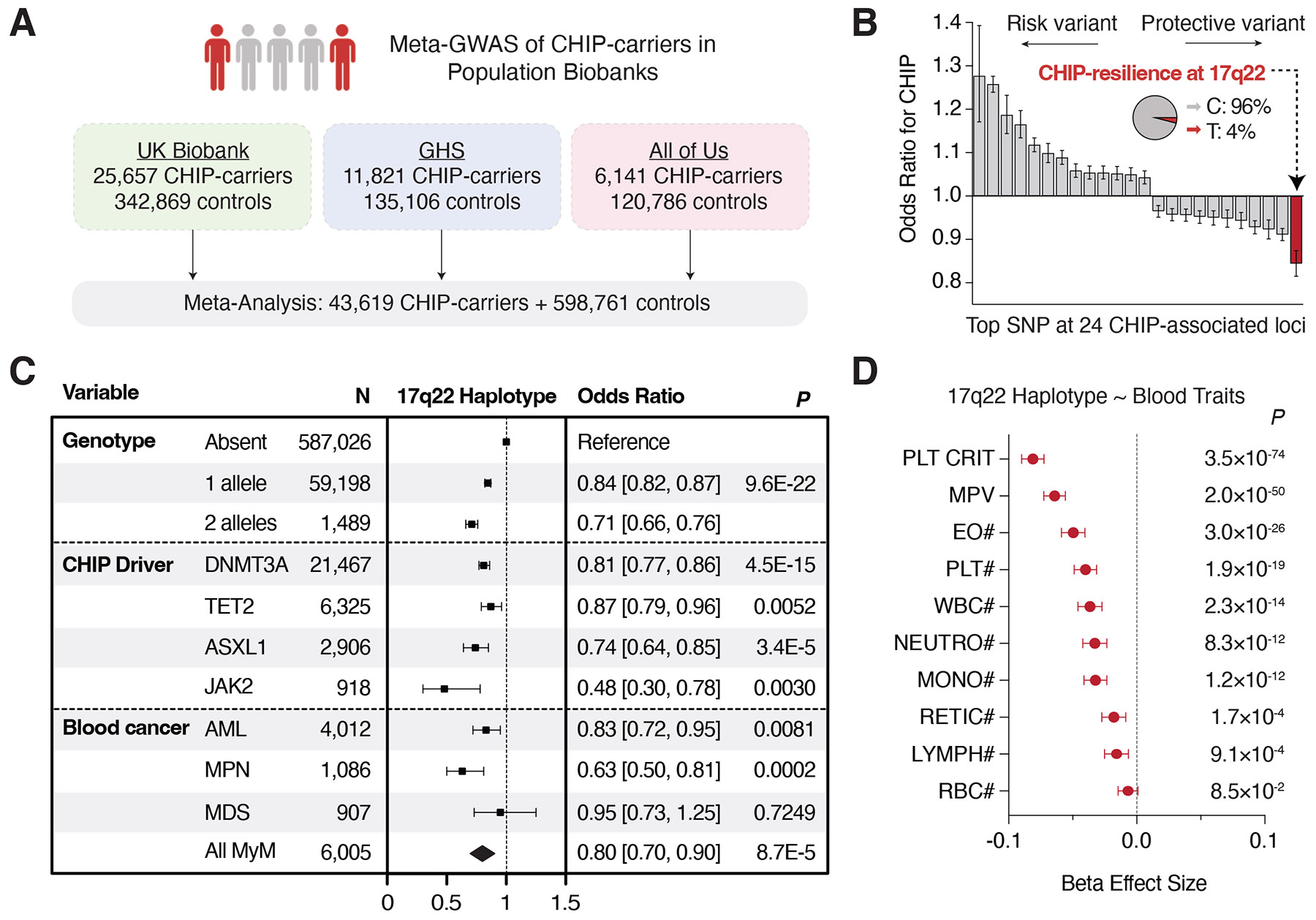
Inherited resilience to CHIP and MyM at the 17q22 locus. (**A**) Approach for meta-analysis of CHIP across UK Biobank, Geisinger Health Study (GHS), and All of Us cohorts. (**B**) Waterfall plot showing meta-analysis odds ratio effect sizes (95% CI) for the most significant variant at 24 CHIP-associated loci. (**C**) Protective effect of 17q22 haplotype for CHIP by number of alleles (top) and per-allele effects by driver mutation (middle) or for MyMs (bottom). MPN = myeloproliferative neoplasm. (**D**) Per-allele effect sizes (beta coefficients) for associations between CHIP-protective 17q22 haplotype and blood cell traits. #, count; PLT CRIT, plateletcrit; MPV, mean platelet volume; EO, eosinophil; PLT, platelet; WBC, white blood cell; NEUTRO, neutrophil, MONO, monocyte; RETIC, reticulocyte; LYMPH, lymphocyte; RBC, red blood cell. Error bars represent mean ± 95% CI.

Given population variability in the expansion of mutant CH clones (3, 21-23), we hypothesized that there may be inherited mechanisms protecting individuals from developing a large clone size. To explore this, we conducted a GWAS meta-analysis for CHIP. We replicated associations at 24 loci reported previously (in UK Biobank and the Geisinger Health Study) (11) in the All of Us cohort and conducted a meta-analysis of 43,619 CHIP-carriers and 598,761 controls (Fig. 1A and table S1). This analysis identified a CHIP-protective haplotype at the 17q22 locus—that is common across populations (fig. S1, A and B)—as the most protective effect [additive odds ratio (OR) = 0.84, 95% confidence interval (CI) = 0.82 to 0.87, *P* = 9.6 × 10^−22^] (Fig. 1B), with homozygotes carrying an ~30% reduced risk of CHIP (Fig. 1C, **top**). An independent GWAS (26) had replicated this CHIP-resilience haplotype (fig. S1, C and D), but the underlying mechanisms have remained unexplored.

In a meta-analysis, the CHIP-protective haplotype was associated with global protection from CHIP subtypes and MyMs. In contrast to variation at other loci with opposing and mutation-selective effects [such as at *TCL1A* (17) and *CD164* (11)], the 17q22 haplotype was associated with consistent resilience to canonical CHIP driver mutations, including *DNMT3A*-CHIP (OR = 0.81), *TET2*-CHIP (OR = 0.87), *ASXL1*-CHIP (OR = 0.74), and *JAK2*-CHIP (OR = 0.48) (Fig. 1C, **middle, and** table S2). Furthermore, carriers had a reduced risk of MyMs (OR = 0.80, 95% CI = 0.70 to 0.90), including myeloproliferative neoplasms (OR = 0.63, 95% CI = 0.50 to 0.81) and acute myeloid leukemia (AML) (OR = 0.83, 95% CI = 0.72 to 0.95) (Fig. 1C, **bottom**), and a phenome-wide association study strongly associated this haplotype with reduced peripheral counts of several blood cell lineages (Fig. 1D) (11). No other phenotypic associations were identified (fig. S1E), suggesting that the CHIP-resilience 17q22 haplotype might selectively alter hematopoiesis to confer a pan-protective effect against CHIP and MyMs.

## rs17834140-T protects from CHIP through HSC-selective down-regulation of MSI2

**Fig. 2.**
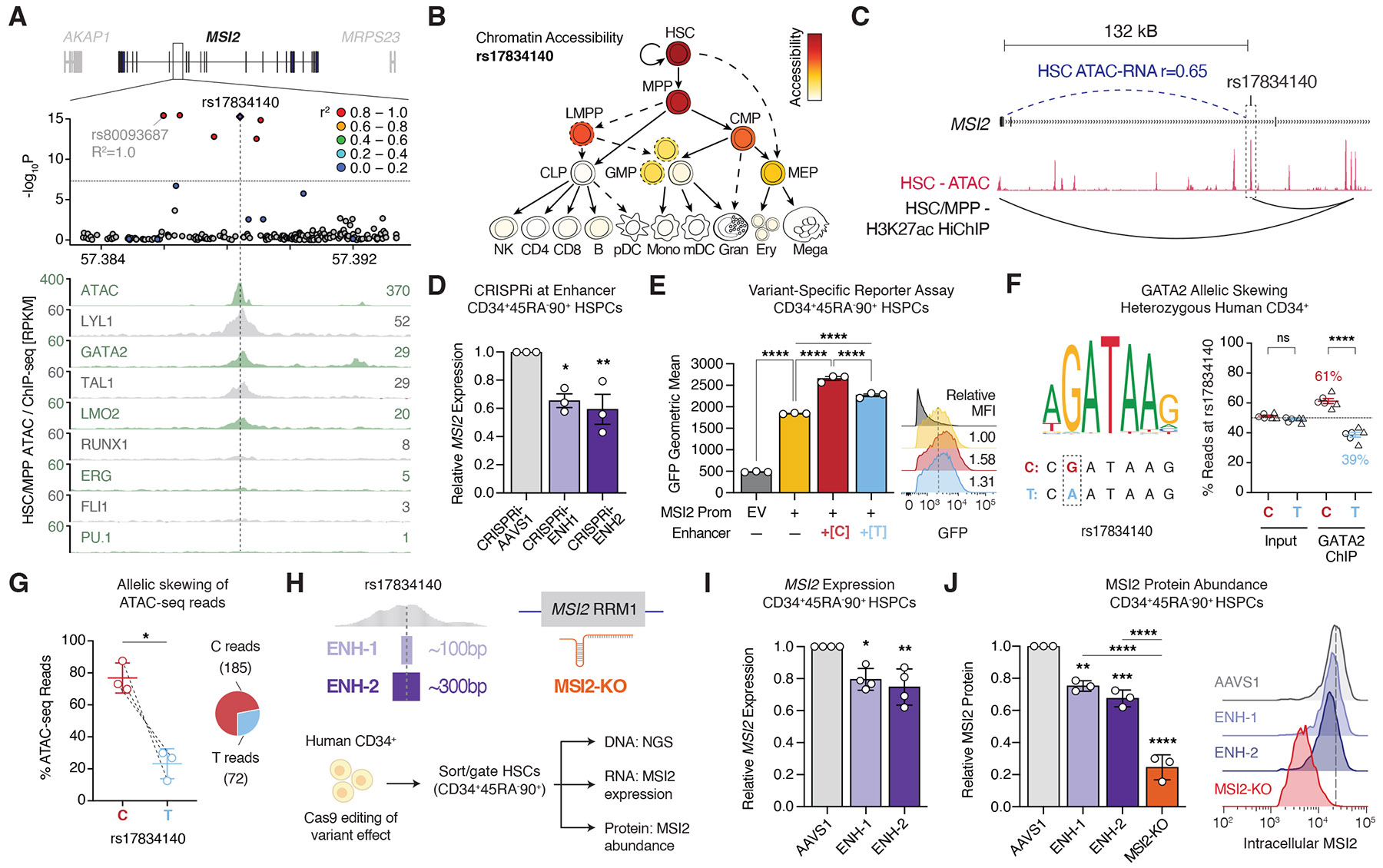
Regulatory variant rs17834140-T protects from CHIP through HSC-selective down-regulation of *MSI2*. (**A**) LocusZoom plot identifying rs17834140 at the 17q22 CHIP-resilience haplotype as a putative causal regulatory variant, with tracks for chromatin accessibility (ATAC) and transcription factor binding (ChIP-seq) in primary human HSPCs. Normalized reads per kilobase of transcript per million (RPKM) counts at rs17834140 are shown on the right side. (**B**) Normalized chromatin accessibility of the rs17834140-harboring regulatory element in hematopoietic cells, indicating HSC-selective regulatory activity. CLP, common lymphoid progenitor; CMP, common myeloid progenitor; GMP, granulocyte-macrophage progenitor; LMPP, lymphoid-primed multipotent progenitor; MEP, megakaryocyte-erythroid progenitor; MPP, multipotent progenitor. (**C**) H3K27ac-HiChIP track showing physical contact between rs17834140 and the *MSI2* promoter in human HSPCs (29). (**D**) Reduced *MSI2* mRNA expression in CD34^+^CD45RA^−^CD90^+^ HSC-enriched cells, 3 days after CRISPR interference targeting the rs17834140-harboring regulatory element. *n* = 3. (**E**) Reporter assay showing enhancer activity of rs17834140 wild-type (C) or CHIP-resilience (T) alleles in CD34^+^CD45RA^−^CD90^+^ HSC-enriched cells, with relative geometric mean fluorescence intensity (MFI) relative to *MSI2*-promoter alone on the right. *N*=3. EV, empty vector. (**F**) ChIP showing reduced GATA2 binding at rs17834140 in primary CD34^+^ HSPCs, across *n* = 2 heterozygous donors (marked as triangles or circles). **(G)** Allele-specific ATAC-seq reads at rs17834140 in HSPCs showing reduced chromatin accessibility with the resilience (T) allele. *n* = 3. (**H**) Editing strategy modeling rs17834140-T effect with ~100-bp (ENH-1) or ~300-bp (ENH-2) microdeletions, or MSI2-KO. NGS, next-generation sequencing. (**I**) Reduced *MSI2* mRNA expression in CD34^+^CD45RA^−^CD90^+^ HSC-enriched cells, 3 days after enhancer microdeletions. *n* = 4. (**J**) Flow cytometry showing reduced intracellular MSI2 protein abundance in CD34^+^CD45RA^−^CD90^+^ gated HSC-enriched cells, 3 days after editing. *n* = 3. Error bars represent mean ± SEM. *P < 0.05; **P < 0.01; ***P < 0.001; ****P < 0.0001; ns is not significant.

To investigate the mechanisms underlying inherited CHIP-resilience, we fine-mapped the 17q22 haplotype. The sentinel single-nucleotide polymorphism (SNP) rs80093687 tagged six fine-mapped SNPs in perfect linkage disequilibrium (*r*^2^ = 1.0) (Fig. 2A, **top**). Although no candidate variants mapped to coding regions or were predicted to alter splicing (table S3), we identified rs17834140-T as a likely causal variant, which was compelling for several reasons. First, rs17834140-T resided within a putative regulatory element bound by a complex of transcription factors critical for maintaining human HSCs (Fig. 2A, **bottom**) (27, 28). Second, the element was selectively accessible in HSCs (Fig. 2B) but not in more differentiated progenitors or mature blood cells, or in hematopoietic cell lines (fig. S2A), suggesting a stem cell-specific regulatory function. Third, H3K27ac-HiChIP (acetylated histone H3 lysine 27–chromatin precipitation with Hi-C) in human HSPCs (29) showed that the element could physically contact the promoter of its proximal gene, *MSI2* (Fig. 2C), which encodes MSI2, an RNA-binding protein that is critical for the maintenance of HSCs (30, 31) and that promotes leukemia and other cancers (32). *MSI2* was the only gene appreciably expressed within 1 Mb of rs17834140 in human HSCs (fig. S2B), and there was high correlation between variant accessibility [by ATAC-seq (assay for transposable-accessible chromatin-sequencing)] and *MSI2* expression across human hematopoietic cells (ATAC-RNA *r* = 0.65) (fig. S2C). CRISPR interference with dCas9-KRAB–mediated repression of the regulatory element reduced *MSI2* expression in CD34^+^CD45RA^−^CD90^+^ HSC-enriched populations (Fig. 2D). These findings suggest that the rs17834140-harboring regulatory element acts as an endogenous enhancer of *MSI2*.

To decipher variant effects, we introduced the rs17834140-harboring regulatory element upstream of a *MSI2* promoter–driven green fluorescent protein (GFP) reporter in primary human HSPCs (fig. S2D). Within transduced CD34^+^CD45RA^−^CD90^+^ cells, addition of the wild-type (rs17834140-C) element augmented reporter expression, validating that this element acts as an enhancer in HSCs (Fig. 2E). Mutagenesis to recreate the CHIP-resilience variant (rs17834140-T) alone in otherwise matched constructs reduced the additional enhancer activity relative to the *MSI2* promoter alone by ~2-fold. We observed that rs17834140-T was predicted to break a highly conserved GATA-binding site [combined annotation dependent depletion (CADD) score: 15.6] (Fig. 2F, **left**), which was notable given that GATA2 binds strongly at this enhancer (fig. S2E). In heterozygous donors, GATA2 chromatin immunoprecipitation (ChIP)–polymerase chain reaction (PCR) at rs17834140 demonstrated ~1.6-fold enrichment of GATA2 binding at the wild-type (C) allele over the CHIP-resilience (T) allele (61 vs. 39% reads) (Fig. 2F, **right**). Consistent with this, we observed a depletion of T alleles in ATAC-sequencing reads from HSPCs of heterozygote donors (Fig. 2G). These data demonstrate that rs17834140-T disrupts GATA2 binding and reduces chromatin accessibility, reducing *MSI2* enhancer function in human HSCs.

We next sought to faithfully model resilience variant effects in primary CD34^+^ human HSPCs. We used CRISPR-Cas9 to minimally excise the putative GATA2-binding site with ~100-base pair (bp) (ENH-1) or ~300-bp (ENH-2) microdeletions harboring rs17834140 (Fig. 2H). We benchmarked phenotypes against edits targeting the RNA-recognition motif 1 (RRM-1) domain of MSI2 resulting in inactivation (KO). Three days after editing, we observed a high efficiency of microdeletions (~70 to 80%) and MSI2-KO (~90%) (fig. S2, F to I). Regulatory element microdeletions reduced *MSI2* mRNA expression in CD34^+^CD45RA^−^CD90^+^ HSPCs relative to safe-harbor *AAVS1*-edited controls (Fig. 2I), selectively within molecularly defined HSCs (fig. S2J) with no impact on other genes at the locus (fig. S2K). Intracellular flow cytometry showed concordant decreases in MSI2 protein abundance in CD34^+^CD45RA^−^CD90^+^ HSPCs after ENH-1 (by ~20% versus AAVS1) and ENH-2 (by ~30% versus AAVS1) microdeletions (Fig. 2J). Thus, our functional analyses suggest that rs17834140-T protects from CHIP through HSC-selective downregulation of MSI2.

## Genetic variation–driven loss of *MSI2* enhancer reduces human HSC fitness

**Fig. 3.**
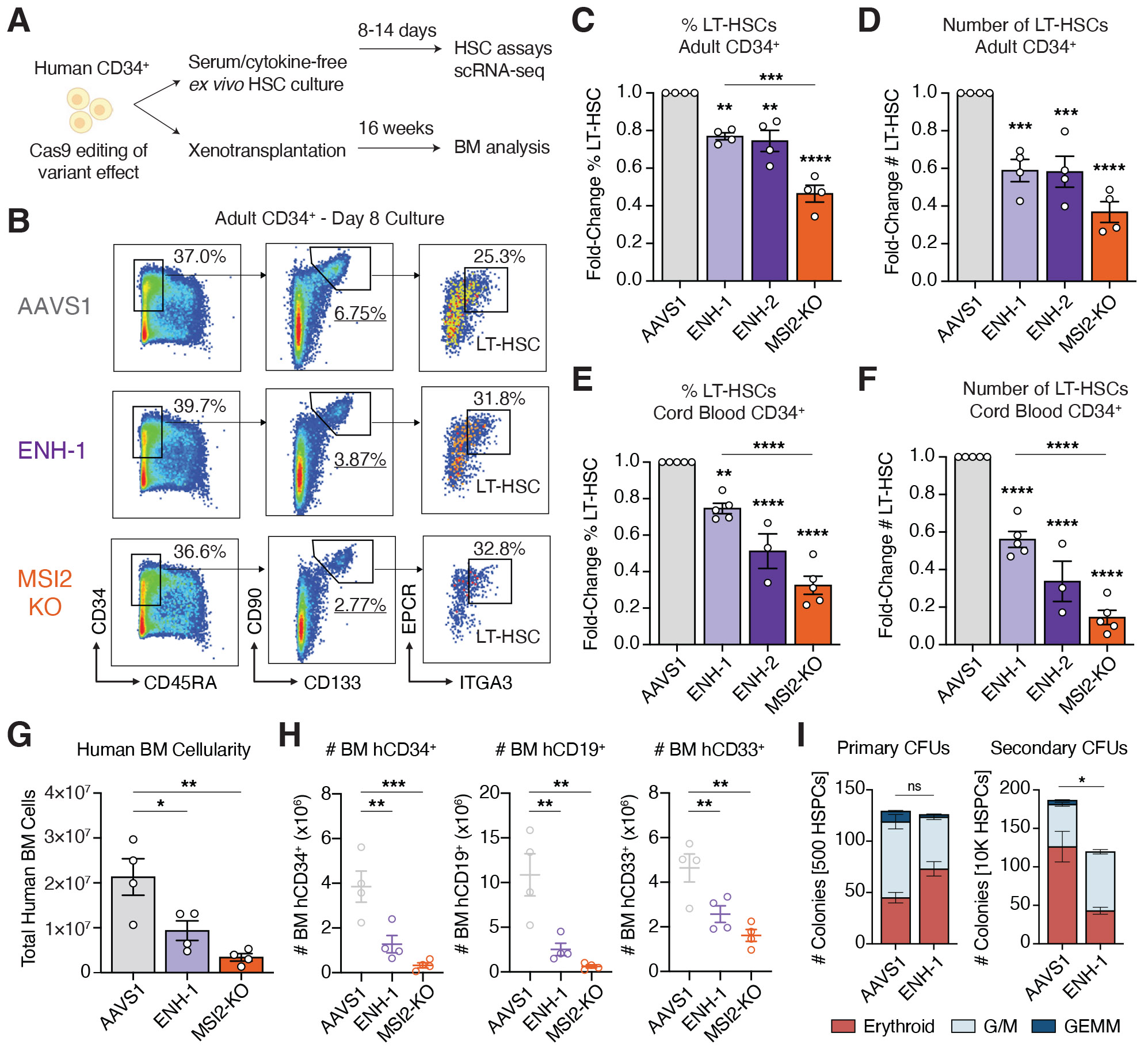
Genetic variation–driven loss of MSI2 enhancer reduces human HSC fitness. (**A**) Outline of HSC assays used to assess CHIP-resilience variant effect. (**B**) Representative flow cytometry gating for phenotypic LT-HSC (CD34^+^CD45RA^−^CD90^+^CD133^+^EPCR^+^ITGA3^+^) immunophenotyping, 6 days after editing adult CD34^+^ HSPCs. (**C**) Proportion of LT-HSCs on day 8 of serum-free adult CD34^+^ cell culture. *n* = 4. (**D**) Total numbers of LT-HSCs on day 8 of serum-free adult CD34^+^ cell culture. *n* = 4. (**E**) Proportion of LT-HSCs on day 14 of cytokine-free cord blood CD34^+^ cell culture. *n* = 3 to 5. (**F**) Total numbers of LT-HSCs on day 14 of cytokine-free cord blood CD34^+^ cell culture. *n* = 3 to 5. (**G**) Total human cells achieving long-term engraftment in bone marrow BM of NBSGW mice, 16 weeks after transplantation with AAVS1-, ENH-1– or MSI2-KO–edited cord blood HSPCs. *n* = 12 mice. (**H**) Total human CD45^+^ engrafted cells in BM expressing lineage markers (CD34^+^ HSPCs, CD19^+^ B cells, CD33^+^ myeloid cells). *n* = 12 mice. **(I)** Colony-forming units (CFUs) formed in primary plating (from 500 HSPCs) and secondary replating (from 10,000 HSPCs), showing loss of self-renewal capacity in ENH-1–edited cells. G/M, granulocyte-macrophage; GEMM, granulocyte-erythrocyte-monocyte-megakaryocyte. *N* = 3. Error bars represent mean ± SEM. *P < 0.05; **P < 0.01; ***P < 0.001; ****P < 0.0001; ns is not significant.

We next sought to understand how inherited CHIP resilience affects human HSCs (Fig. 3A). Six days after editing, MSI2-KO did not affect the proportion of CD34^+^CD45RA^−^ cells in culture, but reduced phenotypic long-term HSCs (LT-HSCs) (CD34^+^CD45RA^−^CD90^+^CD133^+^EPCR^+^ITGA3^+^) principally through loss of primitive CD90^+^ HSPCs (Fig. 3B), consistent with the role of MSI2 in HSC maintenance (30-33). ENH-1 and ENH-2 microdeletions of the rs17834140-site partially phenocopied this effect, in both proportion (Fig. 3C) and total number (Fig. 3D and fig. S3A) of LT-HSCs. In an orthogonal model, we used cytokine-free and chemically defined cultures of human cord blood CD34^+^ HSCs (34). In these conditions, after 14 days, we further observed loss of LT-HSC maintenance (Fig. 3E), with total LT-HSC numbers reduced after ENH-1 (by 44% versus AAVS1) or MSI2-KO (by 85% versus AAVS1) editing (Fig. 3F and fig. S3B). Collectively, these data suggest that loss of *MSI2* enhancer function through genetic variation might alter LT-HSC maintenance.

To determine whether the rs17834140-T-harboring regulatory element could modulate in vivo self-renewal and HSC function, we performed xenotransplantation experiments involving NBSGW (Kit-mutant and immunodeficient NOD.Cg-Kit^W41J^Tyr^+^ Prkdc^scid^Il2rg^tm1Wjl^/ThomJ) mice (35, 36). Mice transplanted with ENH-1–edited human cord blood–derived CD34^+^ cells had reduced total human bone marrow (BM) cellularity (Fig. 3G) and multilineage engraftment of CD34^+^ HSPCs, CD19^+^ B cells, and CD33^+^ myeloid cells (Fig. 3H and fig. S3C) at 16 weeks after transplantation, partially phenocopying a more severe defect in BM reconstitution with MSI2-KO–edited cells. Among engrafted human cells, ENH-1– or MSI2-KO–edited cells had reduced proportions of CD34^+^ HSPCs and CD19^+^ B cells in BM and spleen (fig. S3D), representing a loss of HSCs with multilineage potential. There was a greater percentage of CD33^+^ myeloid cells (fig. S3D) and myeloid colony formation from human CD34^+^ cells harvested from BM (fig. S3, E and F), suggestive of increased myeloid lineage commitment in vivo, and consistent with the role of MSI2 in promoting symmetric self-renewal over asymmetric differentiative divisions (32, 37). Additionally, ENH-1–edited HSPCs had loss of serial replating potential (Fig. 3I), consistent with reduced self-renewal. These data support the idea that CHIP-resilient HSCs have a greater propensity for differentiation at the expense of self-renewal capacity.

## MSI2 stem cell RNA network protects HSCs from CHIP and MyMs

**Fig. 4.**
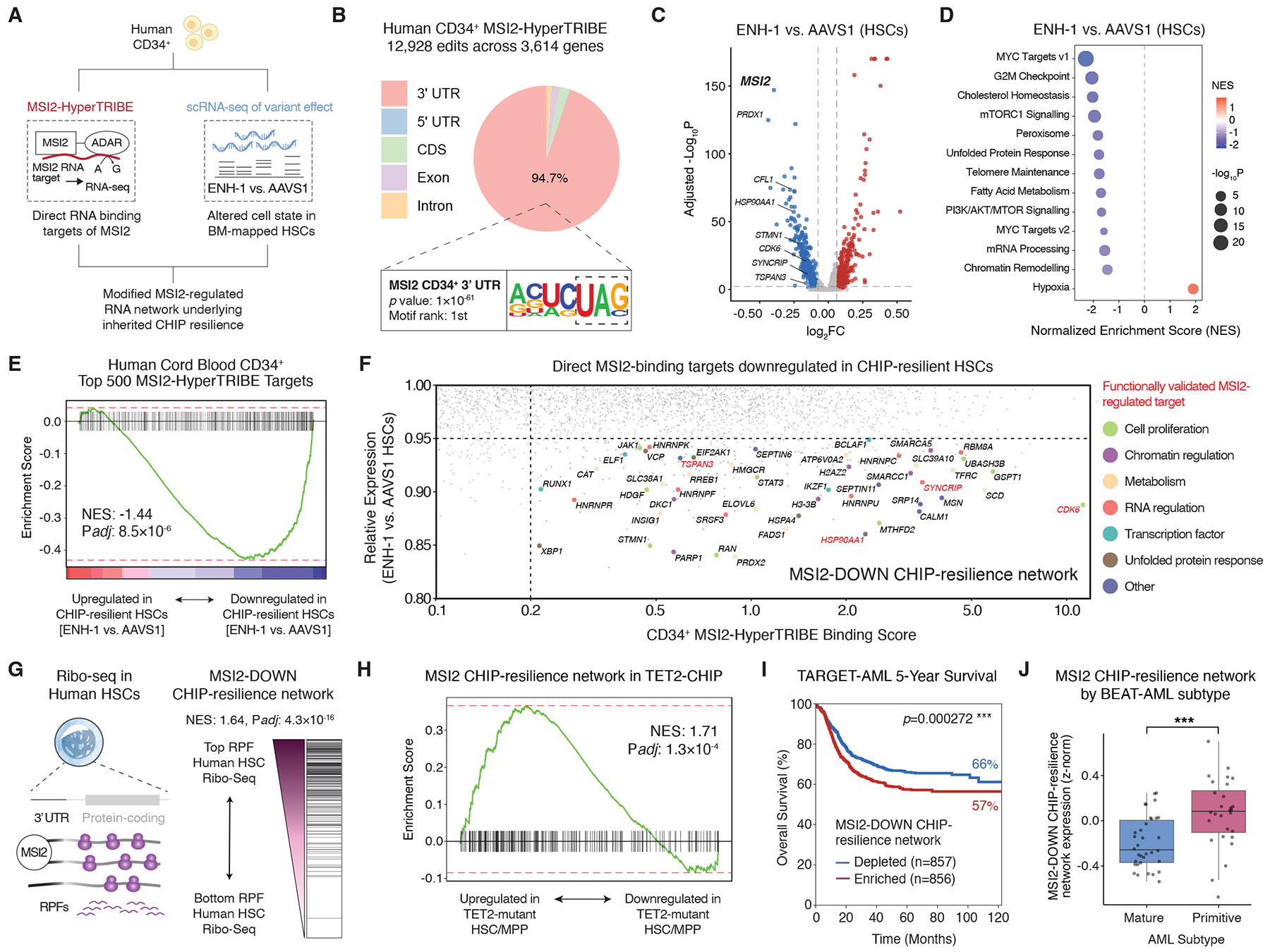
Modified RNA regulation underlying CHIP-resilience in human HSCs. (**A**) Overview of approach to determine MSI2-regulated RNA network in human HSCs. (**B**) MSI2-HyperTRIBE edit sites are enriched in the 3’ UTR of target mRNAs with the UAG MSI2-recognition motif. (**C**) Volcano plot of differentially expressed genes (DEGs) between ENH-1– versus AAVS1-edited HSCs. (**D**) Gene set enrichment analysis of ENH-1– versus AAVS1-edited HSCs. (**E**) CHIP-resilient HSCs (ENH-1 versus AAVS1) show negative enrichment for CD34^+^ MSI2-HyperTRIBE mRNA targets. (**F**) Identification of an RNA network that is MSI2-bound and functionally down-regulated in CHIP-resilient HSCs. **(G)** Ribosome sequencing (Ribo-seq) profiling of human HSCs showing that CHIP-resilience network mRNAs are enriched for ribosome protected fragments (RPFs). (**H**) *TET2* mutant HSC and MPPs show positive enrichment for the MSI2-DOWN CHIP-resilience network, relative to nonmutant cells from the same donors. (**I**) Overall survival of pediatric patients with AML in the TARGET-AML cohort, stratified by median expression of the MSI2-DOWN CHIP-resilience network. **(J)** Expression of the MSI2-DOWN CHIP-resilience network in BEAT-AML diagnostic BM samples, stratified by predominantly mature versus primitive cellular profiles. The center line represents the median, box limits are upper and lower quartiles, and whiskers indicate 1.5× the interquartile range. ***P < 0.001.

Our functional studies led us to investigate mechanisms by which genetic variation–driven down-regulation of MSI2 reduces HSC fitness and protects from CHIP. Because MSI2 is an RNA-binding protein that regulates stability and translation (38) of mRNAs, we sought to define its direct binding targets. Because prior studies were conducted in cell lines (33) or mouse cells (38), the MSI2-regulated network in human HSCs has remained undefined. We therefore used MSI2-HyperTRIBE (38) (Fig. 4A), in which a fusion MSI2-ADAR (RNA-editing enzyme) protein catalyzes A>G edits on MSI2-bound RNA targets, in human cord blood–derived CD34^+^ HSPCs. MSI2-HyperTRIBE identified 12,928 edits across 3614 genes (fig. S4, A to C, and table S4). MSI2-bound mRNAs were enriched for the UAG motif in their 3’ untranslated regions (UTRs) (Fig. 4B), consistent with prior reports (33, 38), and there was strong concordance in bound mRNAs with orthogonal cross-linking and immunoprecipitation sequencing (CLIP-seq) targets of MSI2 (33) (fig. S4, D and E).

We then investigated how reduced MSI2 levels affect its network to confer HSC resilience to CHIP. Single-cell RNA sequencing (scRNA-seq) was performed in human CD34^+^CD45RA^−^CD90^+^ cells with AAVS1, ENH-1, or MSI2-KO edits, and analyses were focused on molecularly defined HSCs (fig. S4, F and G). CHIP-resilient HSCs (ENH-1 versus AAVS1) had a 24% loss of *MSI2* expression and 1005 differentially expressed genes (Fig. 4C and table S5) with high concordance to the MSI2-KO signature (Pearson’s *r* = 0.75) (fig. S4H), representing pathways that include down-regulation of proliferation, MYC targets, and cholesterol biosynthesis genes (Fig. 4D). There was strong negative enrichment of MSI2-HyperTRIBE targets in ENH-1–microdeleted HSCs with reduced MSI2, suggesting that most MSI2-bound mRNA targets were down-regulated in CHIP-resilient HSCs (Fig. 4E).

To understand direct MSI2-bound targets that are functionally altered in human HSCs, we next overlapped MSI2-HyperTRIBE and scRNA-seq datasets. More highly downregulated genes (by ≥5% in ENH-1 versus AAVS1) and edited MSI2 binding targets (mRNAs with ≥20% editing by MSI2-ADAR) were selected. This identified a network of 208 direct MSI2-binding targets that were also down-regulated in ENH-1– versus AAVS1–edited HSCs, hereafter termed MSI2-DOWN genes (Fig. 4F and table S6), whose expression was positively correlated with interindividual variation in *MSI2* levels in HSCs among 131 donors (fig. S4I) (39). This network was enriched for functional MSI2 targets [such as *TSPAN3* (40), *HSP90AA1* (33), *SYNCRIP* (41), and *CDK6* (42)], validating our approach. In addition, the MSI2 network revealed positive regulators of human HSCs [including *TFRC* (43, 44), *DKC1* (45), and *H3-3B* (46)] with no previously studied role in CHIP, nominating candidate regulators of stem cell clonality downstream of MSI2. We leveraged ribosome profiling in primary human CD34^+^CD45RA^−^CD90^+^ HSCs (36) and found that the transcripts of MSI2-DOWN CHIP-resilience network genes were enriched for higher numbers of ribosome protected fragments in comparison to other transcripts (Fig. 4G). This indicated that the RNA network positively regulated by MSI2 is among the more highly translated mRNAs in human HSCs, validating its functionality.

Having identified an RNA network underlying CHIP resilience in HSCs, we sought to understand its clinical relevance. We leveraged TARGET-seq (joint single-cell genotyping and RNA-seq) data of CHIP mutant versus wild-type HSCs from the same donors, enabling insight into the transcriptional impact of CHIP mutations in an isogenic setting (47). The MSI2-DOWN network was up-regulated in *TET2* mutant HSCs in comparison to their wild-type counterparts (Fig. 4H). Furthermore, the MSI2-DOWN network was up-regulated in *ASXL1* mutant versus nonmutant HSPCs of patients with chronic myelomonocytic leukemia (fig. S4J) (48), and ENH-1–edited HSCs were negatively enriched for functional dependencies of *Dnmt3a*^R882H/+^ mutant HSPCs (fig. S4K) (49). To explore the prognostic value of the MSI2-DOWN network in MyMs, we interrogated the TARGET-AML and BEAT-AML cohorts (50, 51). Elevated expression of either *MSI2* or its downstream network at diagnosis predicted a poorer prognosis in pediatric AML (Fig. 4I and fig. S4L) and was further associated with a more primitive and therapy-resistant adult AML subtype (Fig. 4J and fig. S4M). Collectively, we show that the MSI2-DOWN CHIP-resilience network is enriched for positive regulators of HSC proliferation that may promote stem cell clonal advantage in CHIP and MyMs, suggesting that inherited down-regulation of this network might neutralize the impact of acquired CHIP mutations.

## MSI2 levels modify clonal dominance of *ASXL1*-mutant HSCs

**Fig. 5.**
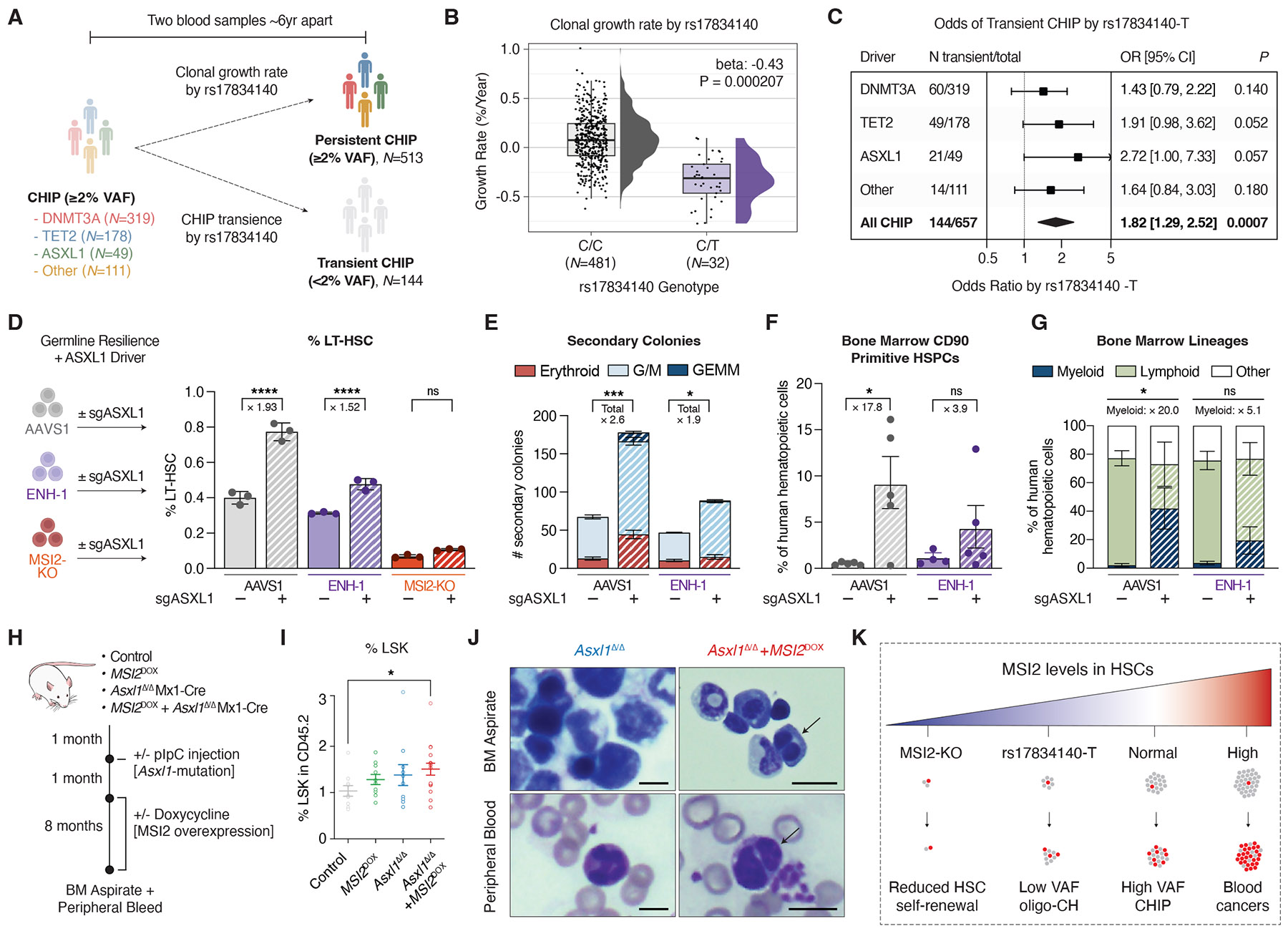
Stem cell MSI2 levels modify clonal dominance of *ASXL1*-mutant HSCs. **(A**) Approach to assess associations between rs17834140-T and longitudinal CHIP progression in a cohort with serial blood samples ~6 years apart. (**B**) Growth rate (% VAF/year) of CHIP-mutations stratified by C/C (*n* = 481) or C/T (*n* = 32) genotype at rs17834140. The center line represents the median, box limits are upper and lower quartiles, and whiskers indicate 1.5× the interquartile range. (**C**) Odds of CHIP transience (≥2% VAF at baseline, <2% VAF at follow-up) by rs17834140-T for driver mutations. (**D**) Dual modeling of germline CHIP-resilience (ENH-1 microdeletion, MSI2-KO, AAVS1 control) with *ASXL1* mutation in primary adult CD34^+^ HSPCs, quantifying LT-HSCs 7 days afer editing. *N* = 3. (**E**) Colony-forming units formed after secondary replating, showing that ENH-1 editing attenuates expansion induced through *ASXL1* mutation. G/M, granulocyte-macrophage; GEMM, granulocyte-erythroid-monocyte-megakaryocyte. *N* = 3. **(F)** Proportions of engrafted human cells expressing CD90 in BM of NBSGW mice after xenotransplantation. *n* = 19 mice. **(G)** Proportions of human engrafted cells expressing myeloid (CD33) or lymphoid (CD19 or CD3) lineage markers in BM of NBSGW mice after xenotransplantation. *n* = 19 mice. **(H)** Workflow of murine model used to evaluate MSI2 and Asxl1 cooperativity. (**I**) Quantification of LSK cells, 1 month after doxycycline treatment in murine model. *n* = 47 mice. (**J**) Representative smears from BM and peripheral blood; arrows indicate MDS features (hyposegmented Pelger-Huët-like neutrophils and binucleate erythroid precursors) in *Asxl1*^Δ/Δ^+*MSI2*^DOX^ mice. Scale bars are 20 μm. (**K**) Model of how MSI2 levels in HSCs regulate stem cell pool size (gray) and clonal advantage of somatic CHIP driver mutations (red). Error bars represent mean ± SEM. *P < 0.05; ***P < 0.001; ****P < 0.0001; ns is not significant.

Finally, we sought to understand how altered MSI2 levels in human HSCs affect the progression of CHIP by analyzing a cohort with targeted sequencing of two serial blood samples taken ~6 years apart (Fig. 5A). Among 513 individuals with persistent CHIP [second variant allele frequency (VAF) also ≥2%], rs17834140-T carriers had a significantly slower measured growth rate of mutant CHIP clones (*P* < 0.001), with many clones showing regression or minimal growth (median growth rates: C/C = 0.12%/year, C/T = –0.31%/year) (Fig. 5B). Indeed, rs17834140-T carriers had greater odds of their CHIP mutation being transient (second VAF <2%) (OR = 1.82, 95% CI = 1.29 to 2.52, *P* = 0.0007) (Fig. 5C), which was most pronounced for *ASXL1*-CHIP (OR = 2.72, 95% CI = 1.00 to 7.33, *P* = 0.057). Together, these data suggest that rs17834140-T attenuates CHIP progression.

To validate MSI2 levels as a modifier of CHIP, we focused on *ASXL1*-mutant CHIP, a common driver in ~20% of myelodysplastic syndrome (MDS) and AML cases, and for which rs17834140-T carried among the most protective associations. We modeled patient *ASXL1* mutations (fig. S5A) in primary human CD34^+^ HSPCs using an exon 12–targeting guide RNA that increases phenotypic LT-HSC maintenance in culture (fig. S5B) and recapitulates clonal expansion in a xenograft model (52). HSPCs were edited to model germline CHIP resilience (80 to 100% editing) and acquired *ASXL1* mutations (91 to 95% editing), with most alleles coedited at both loci (fig. S5C). Whereas *ASXL1* editing expanded LT-HSCs in ex vivo cultures by 1.9-fold, a background of ENH-1 or MSI2-KO editing reduced or abolished the net fitness advantage, respectively (Fig. 5D), and reversed an activated HSC signature induced by *ASXL1* mutation (fig. S5, D and E). Consistent with this, ENH-1 editing reduced *ASXL1* mutation-driven clonal expansion as assessed by serial colony replating (Fig. 5E and fig. S5F). In vivo, engrafted *ASXL1*-mutant HSPCs displayed expansion of primitive CD90^+^ HSPCs (Fig. 5F), as well as increased CD117/KIT^+^ (fig. S5G) and CD33^+^ cells, consistent with myeloid skewing (Fig. 5G); all of these effects were attenuated under a background of ENH-1 editing. These data show how inherited CHIP resilience that reduces MSI2 attenuates the fitness advantage of acquired *ASXL1* mutations in HSCs.

Conversely, stem cell MSI2 levels are commonly up-regulated in MyMs and drive aggressive disease with poor prognosis (53, 54). Therefore, we generated a mouse model to probe the combined impact of MSI2 overexpression and *Asxl1* mutant CHIP (Fig. 5H and fig. S6A). Whereas MSI2 overexpression or *Asxl1* deletion alone had minimal impact on the frequency of HSC-enriched Lin^−^Sca-1^+^c-Kit^+^ (LSK) cells, mice transplanted with *Asxl1*^Δ/Δ^+*MSI2*^DOX^–expressing cells had a 1.5-fold average increase in LSK cell frequency (Fig. 5I). Over 8 months, whereas *Asxl1*^Δ/Δ^ and *MSI2*^DOX^ mice did not show evidence of hematologic disease, *Asxl1*^Δ/Δ^+*MSI2*^DOX^ mice developed reduced peripheral leukocyte and red blood cell counts, along with other blood trait alterations (fig. S6B), and blood smears showed MDS features that included hyposegmented Pelger-Huët-like neutrophils and binucleate erythroid precursors (Fig. 5J and fig. S6C). Taken together, our data suggest a model in which MSI2 levels in HSCs functionally interact with CHIP and MyM driver mutations by modifying their clonal advantage both before and after somatic mutation acquisition (Fig. 5K).

## Discussion

Genetic variation provides opportunities to uncover natural mechanisms for disease resilience. Notable examples include loss-of-function variants in *CCR5* conferring HIV resistance (55), *PCSK9* protecting against cardiovascular disease (56), and *BCL11A* suppression ameliorating hemoglobin disorders (57, 58), insights that have guided therapeutic development. Although much has been learned about cancer predisposition, inherited cancer resilience is largely unexplored. Because purifying selection and rarity of loss-of-function variants limit discovery (59), we reasoned that large population biobanks may uncover such protective variation. In this study, we identified a protective mechanism against CHIP–an exemplar of somatic mosaicism and precursor to MyMs–through selective genetic down-regulation of the RNA-binding protein MSI2 in HSCs.

Our studies provide critical insight into RNA networks that underlie HSC maintenance and clonal advantage in CHIP. RNA-binding proteins are key posttranscriptional regulators of self-renewal (60), and although previous work established MSI2 as a regulator of HSC numbers in vivo (32, 33, 37), its downstream mechanisms and natural genetic modulation have remained unknown. We mapped an MSI2-regulated network in human HSCs, the systematic perturbation of which is likely to deepen our understanding of mechanisms involved in HSC maintenance and the fitness advantage in CHIP. This may also be of broader relevance in MSI2-driven lymphoid malignancies (42, 61, 62) and solid tumors (63-66).

We show that partial and selective down-regulation of MSI2 in HSCs is adaptive (Fig. 5K), without other apparent adverse implications in variant carriers. Together, our results support two (non-mutually exclusive) models, in which reduced MSI2 levels may (i) reduce the baseline HSC pool (lowering the likelihood of any cell acquiring a CHIP driver mutation) or (ii) attenuate the clonal advantage of drivers once acquired. Our study highlights the potential to target MSI2, through small-molecule inhibition or genome editing at its enhancer, for blood cancer prevention. More broadly, we provide an example of how resilience to cancer can arise through inherited genetic variation, motivating the search for other natural pathways that could be leveraged to prevent or treat malignancy.

## Materials and Methods

### All Of Us and GWAS meta-analysis

All of Us (AoU) is a longitudinal cohort containing short-read whole genome sequencing data (mean depth of 30x). Informed consent is in place for all AoU participants, and the protocol was reviewed by the Institutional Review Board (IRB) of the AoU Research Program. Variant-level QC metrics included QUAL > 60, ExcessHet < 54.69, GQ > 20, DP > 10, AB > 0.2 for heterozygotes. Variants with population-specific allele frequency (AF) > 1% or population-specific allele count (AC) > 100 were selected. Sites with more than 100 distinct alternate alleles were excluded.

Individual-level CHIP calls in AoU were ascertained, as previously described (68). Only samples with matching reported sex and genetically inferred sex were included, and analyses were performed on individuals of European ancestry with no diagnosis of hematological malignancy (using filtering criteria as previously reported (68)). PLINK (v1.9) was used to perform logistic regression with Firth correction to assess associations with CHIP for the most significant sentinel SNP at 24 loci previously reported (11). An inverse variance-weighted fixed-effect meta-analysis was performed across AoU associations and summary statistics in UK Biobank (UKB) and Geisinger Health Study (GHS) cohorts (11). Additionally, the effect of the 17q22 CHIP-resilience haplotype (tagged by sentinel SNP rs80093687) on prevalence of specific CHIP driver mutations (*DNMT3A*, *TET2*, *ASXL1*, *JAK2*) was evaluated as a meta-analysis (across AoU and UKB). All analyses in AoU were adjusted for age, sex at birth and the first 5 genetic principal components as covariates.

For associations with myeloid malignancy (MyM) risk, effect estimates for the 17q22 CHIP-resilience haplotype were extracted from GWAS summary statistics for myeloproliferative neoplasm (MPN) [3,797 cases and 1,152,977 controls (10)], acute myeloid leukemia (AML) [4,018 cases and 10,488 controls (69)] and myelodysplastic syndromes (MDS) [907 cases and 5,604 controls (70)]. Phenome-wide association study (PheWAS) summary statistics were previously reported (11), and plotted using the PheWAS package in R.

### Fine-mapping and variant annotation

Statistical fine-mapping was performed using GWAS summary statistics for CHIP in the UKB European ancestry cohort (11) [GWAS catalogue: GCST90165267], and linkage disequilibrium (LD) matrices for UKB participants of British ancestry (71). Summary statistics were converted from hg38 to hg19 using LiftOver. Variants with minor allele frequency (MAF) > 0.001 within 1.5 Mb of the sentinel SNP rs80093687 (7,012 in total) were fine-mapping using SuSIE (72), CARMA (73) and FINEMAP (74), allowing up to 10 causal variants and reporting 95% credible sets for each method. All methods identified the same six variants (rs80093687, rs188761458, rs199691861, rs118121072, rs17834140, rs150497606) in a single 95% credible set.

Fine-mapped variants and those in strong LD with rs80093687 (R^2^ ≥ 0.8, ± 1.5 Mb) were annotated with genomic context, splicing predictions (using SpliceAI (75)), and chromatin accessibility across 13 hematopoietic cell types (76). Normalized mRNA expression for all genes within 1 Mb of rs17834140 was extracted from a single-cell multiome dataset of human HSCs (old-1 and old-2 donors (4)). For ATAC-RNA correlation, hematopoietic cell types were clustered into *N*=75 ‘metacells’ to address single-cell sparsity, and Pearson’s correlation was computed between mean *MSI2* expression and chromatin accessibility at the rs17834140-harboring regulatory element. H3K27ac-HiChIP and ChIP-seq data (LYL1, GATA2, TAL1, LMO2, RUNX1, ERG, FLI1, PU.1) in CD34^+^CD45RA^−^ HSPCs were previously reported (29). Transcription factor motif analysis was conducted using motifBreakR (v2.16) (77), with HOCOMOCO v11 matrices and a statistical threshold of p<1×10^−3^. Allelic skewing in ATAC-seq data of primary human CD34^+^ HSPCs was assessed in three heterozygotes for rs17834140 (GEO accessions GSE194122, GSE219015 and GSE156733). Raw FASTQ files were aligned to hg38 using bwa (v0.7.18), BAM files were generated with samtools (v1.20), and bcftools mpileup was used to call ‘C’ and ‘T’ alleles at rs17834140.

### Primary cell culture

Primary human CD34^+^ HSPCs from mobilized peripheral blood of healthy donors were obtained from the Fred Hutchinson Cancer Research Center (donor IDs available on GitHub repository, at https://github.com/sankaranlab/chip_resilience). Thawed cells were cultured at 5x10^5^ cells/mL in serum-free StemSpan SFEM II medium (StemCell Technologies) supplemented with 1% L-glutamine (ThermoFisher Scientific, 25-030-081), 1X penicillin/streptomycin (Life Technologies, 15140-122), 1X CC100 (containing the cytokines FLT3L, SCF, IL-3 and IL-6; StemCell Technologies, 02690), 100 ng/mL recombinant thrombopoietin (TPO; PeproTech, 300-18), and 35 nM UM171 (StemCell Technologies).

CD34^+^ HSPCs from cord blood of healthy newborns (Pasquarello Tissue Bank, Dana-Farber Cancer Institute) were purified using the EasySep Human Cord Blood CD34^+^ positive selection kit (StemCell Technologies) according to the manufacturer’s instructions. For cytokine-free culture (34), cord blood-derived CD34^+^ HSPCs were cultured at 7×10^4^ to 1×10^5^ cells/mL in Iscove's Modified Dulbecco's Medium (IMDM; Life Technologies), supplemented with 1% L-glutamine (Thermo Fisher Scientific), 1% penicillin/streptomycin (Life Technologies), 1% insulin-transferrin-selenium-ethanolamine (ITSX; Life Technologies), 1 mg/ml polyvinyl alcohol (PVA; Sigma-Aldrich), 1 μM 740Y-P (MedChemExpress), 0.1 μM butyzamide (MedChemExpress), and 70 nM UM171.

### CRISPR interference

For CRISPR interference, dCas9-KRAB (from Addgene plasmid #220838) was subcloned into the backbone of Addgene plasmid #204472, and in vitro transcription was performed to make purified mRNA encoding dCas9-KRAB. On day 2 of culture, HSPCs were washed two times in DPBS, and resuspended in 20 μL of Lonza P3 buffer with supplement. For each nucleofection, HSPCs were mixed with 2 μg dCas9-KRAB mRNA and total 2 μL sgRNA targeting either the *AAVS1* safe-harbor locus or the rs17834140-harboring regulatory element with independent pairs of sgRNAs [*AAVS1*: 1 μL each of sgAAVS1_g1 and sgAAVS1_g2; CRISPRi-ENH-1: 1 μL each of sgENH-1_g1 and sgENH-1_g2; CRISPRi-ENH-2: 1 μL each of sgENH-2_g1 and sgENH-2_g2 sgRNAs] (all sgRNA sequences detailed in Table S7). HSPCs were electroporated in 20 μL Nucleocuvette strips using the Lonza 4D Nucleofector, with the DS-130 program. Immediately after electroporation, 80 μL of prewarmed media were added to the electroporation cuvette, which was placed in an incubator at 37 °C for 5 mins. Cells were then plated at a density of 5x10^5^ cells/mL in adequate complete media. 72 hrs after nucleofection, HSPCs were sorted to enrich for HSCs (DAPI^neg^CD34^+^CD45RA^−^CD90^+^). Subsequently, total RNA was extracted, cDNA was generated, and real-time quantitative PCR (RT-qPCR) was performed to detect *MSI2* expression (as described below).

### Lentiviral packaging and quantification

Lentiviral reporter constructs (as previously described (78)) carrying the enhancer element with wild-type (C) and resilience (T) alleles of rs17834140 [hg38: chr17:57,388,104-57,388,632] were cloned upstream of the human *MSI2* promoter driving GFP reporter expression. The *MSI2* promoter element was synthesized as a g-block [hg38: chr17:57,256,155-57,256,741] (IDT Technologies). For lentiviral packaging, HEK-293T cells were cultured at 37 °C in DMEM (Life Technologies) supplemented with 10% FBS and 1% penicillin/streptomycin. Cells were plated into 15 cm^2^ plates and grown to ~70% confluency on the day of transfection per lentiviral construct. For each plate, 15 μg of psPAX2 packaging plasmid, 7.5 μg of pMD2.G envelope plasmid and 15 μg of construct were added in the presence of Opti-MEM media (Gibco, 31985-062) and Lipofectamine 3000 Transfection Reagent (Invitrogen, L3000001). Viral supernatants were harvested twice at 48 and 72 hrs post-transfection, and concentrated by ultracentrifugation (24,000 rpm for 2 hrs at 4 °C) in the SW32 rotor of Beckman Coulter ultracentrifuge. After ultracentrifugation, the supernatant was decanted and viral pellets were resuspended in ice cold PBS with 0.1% BSA, and frozen at −80 °C till further use. Lentiviral constructs were titrated using the Lenti-X^™^ Provirus Quantitation Kit (Takara, 631239) in K562 cells (cultured at 37 °C in IMDM supplemented with 10% FBS and 1% penicillin/streptomycin), as per manufacturer’s instructions.

### Reporter assay

For lentiviral transduction, concentrated virus was added to CD34^+^ HSPCs on day 1 of culture at equal titres, in the presence of 8 μg/mL cyclosporin H (Sigma-Aldrich, SML1575). HSPCs were then spinfected at 2,000 rpm for 90 mins at 37 °C. 72 hrs after infection, HSPCs were harvested, washed twice with PBS, and incubated for 30 mins with different fluorescent-labelled antibodies: 1:40 dilution of anti-human CD34 PerCP-Cyanine5.5 (clone 561, BioLegend, #343612), 1:50 dilution of anti-human CD45RA APC/Cy7 (clone HI100, BioLegend, #304128) and 1:100 dilution of anti-human CD90 PE (clone 5E10, BioLegend, #328110). Immunophenotypic analyses were performed to quantify geometric mean fluorescence intensity (MFI) of GFP^+^ transduced cells within CD34^+^CD45RA^−^CD90^+^ gated HSC-enriched cells for each lentiviral construct. Analyses were performed on LSRFortessa (BD Biosciences). Data were analyzed using the FlowJo software.

### GATA2 ChIP

Chromatin Immunoprecipitation (ChIP) was performed on chromatin from 10–15×10^6^ primary human CD34^+^ cells, from mobilized peripheral blood of healthy adults or from cord blood of healthy newborns. On day 5 of serum-free culture, cells were cross-linked with 1% methanol-free formaldehyde (Pierce Life Technologies, 28906) and quenched with glycine. Chromatin was processed according to the iDeal ChIP-seq kit for transcription factors (Diagenode, C01010170) with the following modifications. Lysates were sonicated using an E220 sonicator (Covaris, 500239) in microtube AFA Fiber Pre-Slit Snap-Cap tubes (Covaris, 520045) under these conditions: peak incident power = 165 W, duty factor = 2%, cycles per burst = 200, treatment time = 210 s. Sheared chromatin was immunoprecipitated with 5 μg of either anti-GATA2 antibody (clone EPR2822(2), Abcam, ab109241) or control IgG (clone RIG002B, Diagenode, C15410206), and DNA was eluted in 30 μL nuclease-free water. Validation of GATA2 binding was performed by ChIP-qPCR using primers for: *MSI2* enhancer (forward: TTTCCAAAGACCACTGCGCT, reverse: GAAACACCCCAGGAGCAAGA), or negative control myoglobin exon 2 (Diagenode, C17011006-50).

Allele-specific ChIP at rs17834140 was performed as above using cord blood CD34^+^ cells from two healthy newborn donors confirmed to be heterozygous for rs17834140 (C/T) by Sanger sequencing. Following ChIP, a 96 bp amplicon spanning rs17834140 was obtained by PCR (primers as above) and next-generation sequencing (Premium PCR Sequencing, Plasmidsaurus; Oxford Nanopore Technology) was performed. Raw FASTQ files were aligned to hg38 using bwa (v0.7.18), BAM files were generated with samtools (v1.20), and bcftools mpileup was used to quantify skewing of ‘C’ and ‘T’ alleles at rs17834140.

### CRISPR/Cas9 RNP nucleofection and editing analysis

Electroporation was performed on either day 2 (for CD34^+^ from mobilized peripheral blood of healthy donors) or day 3 (for cord blood CD34^+^ from healthy newborns) of culture, using the Lonza 4D Nucleofector with 20 μL Nucleocuvette strips. The Cas9 ribonucleoprotein (RNP) complexes were prepared by combining 2.1 μL of Lonza P3 primary cell nucleofection reagent (Lonza, V4XP-3032), 1.7 μL of 62μM Alt-R S.p. HiFi Cas9 Nuclease V3 (IDT, 1081061) and total 1.0 μL of 100 μM sgRNA in IDTE pH 7.5 (IDT) [*AAVS1*: 0.5 μL each of sgAAVS1_g1 and sgAAVS1_g2; ENH-1 microdeletion: 0.5 μL each of sgENH-1_g1 and sgENH-1_g2; ENH-2 microdeletion: 0.5 μL each of sgENH-2_g1 and sgENH-2_g2; MSI2-KO: 1 μL of sgMSI2-KO]. HSPCs were washed two times in DPBS, then resuspended in 20 μL of Lonza P3 buffer with supplement (in presence of nucleofection enhancer). Cells were electroporated using the DZ-100 program in a 4D-Nucleofector X Unit (20 μL cuvettes). Immediately after electroporation, 80 μL of prewarmed media were added to the electroporation cuvette, which was placed in an incubator at 37 °C for 5 mins. Cells were then plated at a density of 5x10^5^ cells/mL in adequate complete media.

For analysis of DNA editing, CD34^+^CD45RA^−^CD90^+^ HSC-enriched cells were harvested for genomic DNA extraction at least 72 hrs post-nucleofection. PCR fragments flanking the editing site (at least 250 bp upstream and downstream) were amplified (with primer sequences detailed in Table S7) and editing frequencies and outcomes were detected by either Sanger sequencing (and analyzed with the ICE analysis tool from Synthego) or next-generation sequencing (NGS). For NGS, Premium PCR Sequencing was performed by Plasmidsaurus using Oxford Nanopore Technology with custom analysis and annotation, and analysis was performed using CRISPResso2 (79).

### Real-time quantitative PCR (RT-qPCR) for MSI2 Expression

Total RNA was obtained on day 5 of HSPC culture (72 hrs following CRISPR/Cas9 editing or dCas9-KRAB targeting) using the Total RNA Purification Micro Kit (Norgen, 35300) as per manufacturer’s instructions, including DNase I digestion (Norgen, 25710). 100 to 500 ng of total RNA was used for reverse transcription using the iScript cDNA synthesis (BioRad, # 1708890) or PrimeScript^™^ RT Master Mix (Takara, RR036A). The cDNA product was used for real-time PCR analysis using iQ SYBR green supermix (BioRad, #1708882), with primers specific to *MSI2* or *GAPDH* (sequences detailed in Table S7). Three technical replicates were performed for each sample, and the mean value was selected for further analysis. The relative expression of each target gene was first normalized to *GAPDH* housekeeping gene expression and then represented as fold changes (2^−ddCt^) relative to the indicated control conditions.

### Intracellular flow cytometry

MSI2 intracellular protein abundance was quantified on day 5 of culture (72 hrs after editing). HSPCs were harvested, washed once in DPBS, and incubated for 30 mins with different fluorescent-labelled antibodies: 1:40 dilution of anti-human CD34 PerCP-Cyanine5.5 (clone 561, BioLegend, #343612), 1:50 dilution of anti-human CD45RA APC/Cy7 (clone HI100, BioLegend, #304128) and 1:100 dilution of anti-human CD90 PE (clone 5E10, BioLegend, #328110). Cells were washed, fixed with 4% paraformaldehyde for 15 mins at RT, washed, permeabilized with 0.2% Tween 20 for 15 mins at RT and washed again. Next, cells were then stained with a 1:100 dilution of rabbit anti-MSI2 monoclonal antibody (clone EP1305Y, Abcam, ab76148) for 30 mins at RT, washed, then incubated with a 1:2,000 dilution of goat anti-rabbit AlexaFluor-488 antibody (Invitrogen, A-11034) for 30 mins at RT and washed. Immunophenotypic analyses were performed to quantify the geometric mean fluorescence intensity (MFI) of GFP within CD34^+^CD45RA^−^CD90^+^ gated HSC-enriched cells. Analyses were performed on LSRFortessa (BD Biosciences). Data were analyzed using the FlowJo software.

### Flow cytometry and cell sorting

Immunophenotypic analyses for long-term (LT)-HSCs were performed on adult CD34^+^ HSPCs (on day 8 of serum-free culture, 6 days after editing) or cord blood CD34^+^ HSPCs (on day 14 of cytokine-free culture, 11 days after editing). Cells were harvested, washed and incubated for 30 mins with fluorescent-labelled antibodies: 1:40 dilution of anti-human CD34 PerCP-Cyanine5.5 (clone 561, BioLegend, #343612), 1:50 dilution of anti-human CD45RA AlexaFluor-488 (clone HI100, BioLegend, #304114), 1:100 dilution of anti-human CD90 PE/Cy7 (clone 5E10, BD Biosciences, # 561558), 1:100 dilution of anti-human CD201 (EPCR) PE (clone RCR-401, BioLegend, #351904), 1:33 dilution of anti-human CD49c (ITGA3) APC (clone ASC-1, BioLegend, #343808). Adult-derived HSPCs in serum-free culture were additionally stained with 1:20 dilution of anti-human CD133 Super Bright 436 (clone 7, BioLegend, #372808). All analyses were performed on LSRFortessa (BD Biosciences). Data were analyzed using the FlowJo software. Total LT-HSC numbers were calculated as a product of the frequency of LT-HSCs by flow cytometry and total cell number in culture.

Fluorescence-activated cell sorting (FACS) was conducted to enrich for HSCs, for DNA editing analysis, RT-PCR of *MSI2* expression, and for single-cell and bulk RNA-sequencing experiments. FACSymphony S6 Cell Sorter (BD Biosciences) was used to sort with a 100 μm nozzle for viable cells (negative for DAPI) with surface expression of CD34^+^CD45RA^−^CD90^+^ markers.

### Xenotransplantation and animal models

All animal procedures were performed under a protocol approved by the Boston Children’s Hospital Institutional Animal Care and Use Committee (IACUC). CD34^+^ HSPCs purified from human cord blood of healthy donors were edited on day 2 of culture (as indicated below), and cultured for 48 hr. On day 4 of culture, flow cytometry was used to assess % CD34^+^ in culture for each condition, and 200,000 CD34^+^ cells per mouse were injected via tail vein into Kit-mutant and immunodeficient NOD.Cg-Kit^W41J^Tyr+ Prkdc^scid^Il2rg^tm1Wjl^/ThomJ (NBSGW) mice (JAX#026622). To prevent infections, the mice were provided with autoclaved sulfatrim antibiotic water, which was changed weekly.

For experiments assessing impact of CHIP-resilience on HSC function, HSPCs were edited at AAVS1, ENH-1 or MSI2-KO. Animals were euthanized at 16 weeks post-transplantation, and their bone marrows (BMs) and spleens were collected for analysis. BM cells were obtained by flushing the bilateral femurs and tibias, while spleens were carefully minced. Cell suspensions were counted prior to further analyses. For immunophenotypic analyses, cells were stained with the following antibodies: 1:200 dilution of anti-human CD45 APC (clone HI30, BioLegend, #304037), 1:400 dilution of anti-mouse CD45 FITC (clone 30-F11, BioLegend, #103018), 1:400 dilution of anti-human CD3 BV786 (clone SK7, Fisher Scientific, BDB563800), 1:100 dilution of anti-human CD19 PerCP-Cyanine5.5 (clone HIB19, BD Biosciences, #561295), 1:100 dilution of anti-human CD34 APC-Cyanine7 (clone 561, BioLegend, #343614, 1:100 dilution of anti-human CD33 PE (clone HIM3-4, Invitrogen, #12-0339-42), and 1:100 dilution of anti-human CD15 AlexaFluor700 (clone HI98, BioLegend, #301920).

For experiments assessing how CHIP-resilience modifies the effect of *ASXL1* mutation, HSPCs were editing (as detailed under ‘Modeling human *ASXL1* mutant CHIP’ section below) at: i) sgAAVS1_g1+sgAAVS1_g2; ii) sgAAVS1_g1+sgASXL1-ex12; iii) ENH-1+AAVS1_g2; iv) ENH-1+sgASXL1-ex12). Animals were euthanized at 9 weeks post-transplantation, and their BMs were collected for analysis. BM cells were obtained by flushing the bilateral femurs and tibias. Cell suspensions were counted prior to further analyses. For immunophenotypic analyses of lineages, cells were stained with the following antibodies: 1:200 dilution of anti-human CD45 APC (clone HI30, BioLegend, #304037), 1:400 dilution of anti-mouse CD45 FITC (clone 30-F11, BioLegend, #103018), 1:400 dilution of anti-human CD3 BV786 (clone SK7, Fisher Scientific, BDB563800), 1:100 dilution of anti-human CD19 PE-Cyanine5 (clone HIB19, BD Biosciences, #555414), 1:100 dilution of anti-human CD117 PE (clone 104D2, BioLegend, #313204, 1:100 dilution of anti-human CD33 Super Bright 436 (clone HIM3-4, Invitrogen, #62-0339-42), and 1:100 dilution of anti-human CD11b PE/Cy7 (clone ICRF44, BioLegend, #301322). For immunophenotypic analyses of primitive HSPCs, cells were stained with the following antibodies: 1:200 dilution of anti-human CD45 APC (clone HI30, BioLegend, #304037), 1:40 dilution of anti-human CD34 BV421 (clone 561, BioLegend, #343610), 1:50 dilution of anti-human CD45RA AlexaFluor-488 (clone HI100, BioLegend, #304114), and 1:100 dilution of anti-human CD90 PE (clone 5E10, BioLegend, #328110). Analyses were performed on LSRFortessa (BD Biosciences). Data were analyzed using the FlowJo software.

### Colony-forming unit cell assay

Three days post-nucleofection, edited CD34^+^ HSPCs were plated at a density of 500 cells/mL in methylcellulose medium (H4034, Stem Cell Technologies) according to manufacturer’s instructions. Cultures were incubated for 10 days at 37 °C with 5% CO_2_ prior to colony quantification. For serial replating, cells from primary colonies were harvested and replated at 10,000 cells/mL (equalized between conditions). Secondary colonies were counted after 8-10 days.

For CFU assays from long-term xenotransplant recipients, human CD34^+^ cells were enriched from mouse bone marrow 16 weeks post-transplant using the Human CD34 MicroBead Kit (Miltenyi Biotec, 130-046-702). From each mouse, 5,000 CD34^+^ HSPCs were plated in 1 mL methylcellulose medium (H4434, StemCell Technologies) according to the manufacturer’s instructions, and colonies were counted 14 days post-plating.

### MSI2-HyperTRIBE

CD34^+^ HSPCs from cord blood were purified using the CD34 MicroBead Kit (Miltenyi Biotec, 130-046-703) according to the manufacturer’s instructions. Purified CD34^+^ cells were cultured for 2 days in IMDM supplemented with 20 % BIT 9500 (StemCell Technologies, #09500), 1X penicillin/streptomycin (Corning, 30-003-CI), and cytokines including 100 ng/mL Human SCF (Peprotech, 300-07-10UG), 10 ng/mL hFLT3L (Peprotech, 300-19-10UG), 100 ng/mL hTPO (Peprotech, 300-18-10UG), and 20 ng/mL hIL-6 (Peprotech, 200-06-20UG). Cells were then transduced with high-titer, concentrated retroviral suspensions encoding either MIG (empty vector control) or MSI2-ADAR constructs in the presence of 8 μg/mL polybrene (Millipore, TR1003G), and spinfected at 700 g for 1.5 hrs. A second round of transduction was performed the following day. GFP-positive cells were sorted using a BD FACSAria cell sorter 24 hrs after second transduction. Three independent biological replicates were performed. RNA was extracted from bulk sorted cells using the SMARTer RNA extraction method. cDNA was subjected to automated paired-end library construction for sequencing on an Illumina HiSeq 2000 platform (PE100 read length). Sequencing was performed at a depth of 30–40 million reads per sample.

### MSI2-ADAR editing site calling

Sequencing reads from three MSI2-ADAR and three control (empty vector) samples were aligned to the human T2T reference genome chm13v2.0 (80) using minimap2 (v.2.26) (81). All possible single-nucleotide mismatches were piled up with cellsnp-lite (v1.2.3) (82) in mode 2b in each sample individually, requiring a minimum coverage of 10 and a minor allele frequency of at least 0.01. Mismatches were annotated against the GENCODE v35 GTF to determine their overlap with gene regions. Those annotated as common or rare single-nucleotide polymorphisms (SNPs) in human dbSNP (v155) were excluded from further analysis. Because editing sites in MSI2-ADAR samples may not appear in controls, where they remain unedited and do not appear as mismatches, bcftools (v1.20) (83) was used to extract allele counts for any mismatch present in at least two MSI2-ADAR samples, across all six samples. We retained only A>G mismatches on the forward strand or T>C mismatches on the reverse strand in control samples to ensure that A or T was the major allele in the control.

Allele counts of retained mismatches were modelled using a negative binomial generalized linear model (GLM) with a log link (via MASS::glm.nb). Specifically, for each mismatch i:

$$k_{i}∼\text{NegBinom}(\mu_{i},\theta ),\log(\mu_{i})=\beta_{0}+\beta_{1}{\text{group}}_{i}+\log(n_{i})$$

where $k_{i}$ is the minor allele count, $\mu_{i}$ is the expected count of $k_{i}$, θ is the dispersion parameter estimated by the model, $n_{i}$ is coverage (with $\log(n_{i})$ serving as an offset), and ${\text{group}}_{i}$ indicates whether the sample is MSI2-ADAR or control. P-values of the MSI2-ADAR group coefficient $\beta_{1}$ were adjusted with the Benjamini-Hochberg method. A smaller p-value reflects a larger difference in editing frequency between MSI2-ADAR and control samples. To estimate editing frequency in each group, we regressed the observed edited counts ($k_{i}$) on the total coverage ($n_{i}$) without an intercept: $k_{i}∼0+n_{i}$. Ultimately, we kept mismatches that met all of the following criteria: 1) the difference in editing frequencies between MSI2-ADAR and control samples was ≥0.1; 2) the editing frequency in control samples was <0.02; 3) FDR < 0.05 in the GLM test.

The CD34^+^ MSI2-HyperTRIBE score was calculated by taking the sum of total differential editing frequencies per gene (for instance, if a target mRNA was edited at two separate nucleotides in 50% and 70% of reads, then the total score was 1.2). For orthogonal validation, targets and edit sites were presented for MSI2-CLIP-seq in hematopoietic cells reported previously (33). To identify motifs surrounding MSI2-ADAR editing sites, we used a similar strategy as previously reported (38). Briefly, sequences extending ±100 bp of each editing site in the 3′ UTR were extracted, and overlapping regions were merged. As a background set, 201-bp segments were randomly selected from 3′ UTRs in the reference genome that did not overlap with the target sequence pool. The HOMER2 software (84) was used to search for enriched RNA motifs of length 6, 7, or 8.

### Single-cell RNA sequencing and analysis

Droplet-based digital 3’-end single cell RNA sequencing (scRNA-seq) was performed on a Chromium Single-Cell Controller (10X Genomics) using the Chromium Next GEM Single Cell 3’ Reagent Kit v3.1 according to the manufacturer’s instructions. CD34^+^ HSPCs derived from mobilized peripheral blood of 2 distinct healthy donors were edited at AAVS1, with ENH-1 microdeletion or MSI2-KO, on day 2 of culture. 72 hrs after editing, cells were sorted with a 100 μm nozzle for viable cells (DAPI negative) and CD34^+^CD45RA^−^CD90^+^ cell-surface markers. Approximately 1.6x10^4^ viable cells from each sample were utilized for subsequent scRNA-seq processing, with an estimated recovery of 8,000-12,000 cells per condition. Briefly, single cells were partitioned in Gel Beads in Emulsion (GEMs) and lysed, followed by RNA barcoding, reverse transcription, and PCR amplification (11 cycles). scRNA-Seq libraries were prepared according to the manufacturer’s instructions, checked, and quantified on BioAnalyzer instrument. Sequencing was performed on a Nova Seq S2 (Illumina).

The raw scRNA-seq FASTQ files were processed with the CellRanger (v8.0.1) pipeline to map in the reference genome (GRCh38). We excluded cells with unique molecular identifier (UMI) counts less than 2,000 or greater than 25,000, or mitochondrial UMI fraction higher than 20%, and removed potential doublets by a threshold of doublet score > 0.25 using ScrubletR, which resulted in a total of 57,412 cells for AAVS1 (donor 1 = 10,059 and donor 2 = 12,399), ENH-1 (donor 1 = 7,281 and donor 2 = 8,345), and MSI2-KO (donor 1 = 11,330 and donor 2 = 7,998) edited cells. Symphony R package was used to project the cells on a human BM reference (https://github.com/andygxzeng/BoneMarrowMap) (85), to annotate molecularly defined HSCs. A standard Seurat framework (v4.4.0) was used to conduct normalization, principal component analysis (PCA), and dimensionality reduction. The feature-barcode matrix was normalized by the total read count and log-transformed, and the top 3,000 variable features were selected by the vst method in the FindVariableFeatures function. The normalized expression was scaled by Seurat’s ScaleData function, and ribosomal (RPS/RPL) and mitochondrial (MT-) genes were filtered. PCA was performed using the RunPCA function (npc = 30). The sample-dependent technical variation was corrected by using Harmony (86). Uniform Manifold Approximation and Projection (UMAP) was conducted to reduce dimensions to embed the cells into two-dimensional space. Seurat’s FindMarkers function using “wilcox” method was applied within molecularly defined HSCs to identify differentially expressed genes between ENH-1 vs. AAVS1-edited cells, or MSI2-KO vs. AAVS1-edited cells, with a significance threshold of Benjamini & Hochberg (BH)-adjusted *P* < 0.05, an absolute change in relative expression of ≥ 5%, and minimum percent of expressed cells ≥ 10%. Gene set enrichment analysis was performed using the fGSEA package (https://github.com/ctlab/fgsea/) using the 2024 Hallmark gene sets. Figures were generated using R (v4.4).

### Mapping and validating CHIP-resilience network

The MSI2-DOWN CHIP-resilience network (total 208 genes) was defined by mRNA targets with CD34^+^ MSI2-HyperTRIBE score ≥0.2, as well as significantly downregulated following enhancer perturbation (relative expression ≤0.95 and adjusted *P* < 0.05 in ENH-1 vs. AAVS1 HSCs). Inter-individual variation and correlation between the z-normalized expression of *MSI2* and its network was analysed from a reference single cell dataset of circulating HSCs from healthy donors (39). Ribo-seq data was generated in CD34^+^CD45RA^−^CD90^+^ human HSCs, as previously reported (36); raw counts of ribosome protected fragments (RPFs) were normalized to transcript length, and the mean log-normalized RPKM (reads per kilobase per million mapped reads) across three replicates was ranked, followed by GSEA using the fGSEA package.

To validate clinical relevance of the MSI2-regulated network, TARGET-seq data (targeted high-sensitivity single cell mutational analysis with parallel RNA-seq) for *TET2* mutant vs. non-mutant HSC/MPPs was previously reported (47). The transcriptomic profile of *ASXL1* mutant vs. non-mutant CD34^+^ cells was generated through re-analysis of a previously published RNA-seq dataset (48). The gene set of functional *Dnmt3a*^R882H^ vulnerabilities was previously described (49). Survival analysis in the TARGET-AML dataset was performed using Survival Genie 2.0 (87), within diagnostic BM samples from *N*=1,713 patients (50); overall survival from time of diagnosis was stratified by median normalized expression of *MSI2*, or the downstream MSI2-DOWN CHIP-resilience network (*N*=208 genes), with all other default settings. Expression of *MSI2* or its network was z-normalized from RNA-seq of diagnostic BM samples from the adult BEAT-AML cohort (51), and stratified as either ‘mature’ or ‘primitive’ as previously reported through single cell deconvolution (88).

### Longitudinal clonal growth rate analysis

Targeted, error-corrected sequencing was performed on two serial blood samples from 3,000 people in the Vanderbilt BioVU biobank, with custom-designed probes for 22 CHIP-associated genes, as previously described (89, 90). Vanderbilt University Medical Center’s Institutional Review Board oversees BioVU and approved this project (IRB #201783). UMIs were used for error correction, excluding mutations detected from a single UMI. The mean coverage depth was 1725x after de-duplication. CHIP mutations were called for variants with 100x total read depth, 3 variant allele reads, and variant allele fraction (VAF) ≥ 2% in at least one blood draw. The median time between blood draws was 5.7 years (range: 0.7-13). 657 CHIP mutations (VAF ≥ 2%) were identified at the first time point, of which at the second time point 513 mutations had a VAF ≥ 2% (persistent CHIP) and 144 had a VAF < 2% (transient CHIP). For those with persistent CHIP, growth rate r was modelled with a compound interest formula $r=\frac{{VAF_{2}}^{1∕t}}{VAF_{1}}-1$ where t is the duration in years between blood draws, as previously done (89, 90). In those with CHIP mutations at baseline, rs17834140 genotypes were extracted using GATK’s HaplotypeCaller (91). For persistent CHIP mutations, we modeled growth rate $r∼\text{age}+{\text{age}}^{2}+\text{VAF}+\text{sex}+\text{rs}17834140\text{-}T+\text{driver gene}$ (encoded as a factor), and extracted the coefficient of rs17834140-T. For all CHIP mutations at baseline, we performed logistic regression of transient_CHIP (0/1) ~ age + age^2^ + VAF + sex + rs17834140-T + driver gene (encoded as a factor). We extracted the odds ratio of transient CHIP by exponentiating the coefficient of rs17834140-T. We repeated the models for the DNMT3A, TET2, ASXL1, and other driver genes separately, and removed driver gene as a covariate for these models.

### Modeling human ASXL1 mutant CHIP and bulk RNA-seq

To model germline CHIP-resilience and acquired *ASXL1* mutations, CRISPR/Cas9 was used to edit CD34^+^ cells (from mobilized peripheral blood) on day 1 of culture. HSPCs were edited (as described in previous section) with total 2.0 μL of 100 μM sgRNA, with 1.0 μL to model germline effects [AAVS1: 1.0 μL of sgAAVS1_g1; ENH-1 microdeletion: 0.5 μL each of sgENH-1_g1 and sgENH-1_g2; MSI2-KO: 1 μL of sgMSI2-KO], and 1.0 μL to model somatic mutation [control AAVS1: sgAAVS1_g2; ASXL1-exon 12: sgASXL1-ex12]. Immunophenotyping of LT-HSCs was performed 7 days after editing.

For bulk RNA-sequencing of *ASXL1* edited human HSPCs, FACS was performed on day 8 of culture (7 days after editing) for viable cells (negative for DAPI) and CD34^+^CD45RA^−^CD90^+^ markers to enrich for HSCs. Total RNA was obtained from sorted cells using the Total RNA Purification Micro Kit (Norgen, 35300) as per manufacturer’s instructions. Ultra-low input RNA-seq was conducted with ~30M reads per sample, across 2 independent experiments in edited HSPCs (AAVS1-sg1+AAVS1-sg2, AAVS1-sg1+ASXL1, ENH-1+ASXL1 and MSI2-KO+ASXL1). FASTQ files were processed for quality control using FastQC (v0.12.1) to assess sequence quality. Salmon (v1.5.2) was used to align reads to the reference transcriptome (GRCh38, Ensembl v104), to quantify gene expression levels. Transcript abundance estimates were imported into R for differential expression analysis using DESeq2 (v1.34.0). Gene set enrichment analysis was performed using the GO Biological Process 2021 database, and the activated HSC signature previously reported (43).

### Asxl1-mutant mouse model

We crossed Mx1-Cre^−^ *Asxl1*^f/f^ or Mx1-Cre^+^ *Asxl1*^f/f^ mice with Col-tet on-*MSI2*/ROSA-rTTA mice, to generate Mx1-Cre^−^ *Asxl1*^f/f^ *MSI2*^wt/ht^ (Control), Mx1-Cre^−^ *Asxl1*^f/f^ *MSI2*^ht/ht^ (*MSI2*^DOX^), Mx1-Cre^+^ *Asxl1*^f/f^ *MSI2* ^wt/ht^ (*Asxl1*^f/f^) and Mx1-Cre^+^ *Asxl1*^f/f^ *MSI2*^ht/ht^ (*Asxl1*^f/f^+*MSI2*^DOX^) mice. 1 million whole BM cells from donor plus 200K CD45.1 helper BM cells were transplanted into lethally irradiated B6-CD45.1 recipient mice. Depletion of *Asxl1* was initiated one-month post-transplantation by administering three intraperitoneal injections of pIpC HMW (InVivogen, vac-pic) at a dose of 10 mg/kg. MSI2 overexpression was induced by administration of 2 mg/mL Doxycycline hyclate (Fisher Scientific, AC446060050) supplemented with 10 mg/mL sucrose in the drinking water, starting from one month after pIpC treatment. Peripheral blood or BM cells were collected at designated time points and analyzed by flow cytometry.

### Quantification and statistical analysis

In all experiments, data were presented as mean ± standard error of mean. When comparing two samples, a two-tailed Student’s t-test was used to test statistical significance. When one of the two samples was a default value (as in fold change comparison), the one sample t- and Wilcoxon test was applied. When comparing three or more samples, Levene’s test was first used to test equality of variance. If the variance across all samples were tested insignificantly differed, one-way or two-way ANOVA with Dunnett’s test (for multiple comparisons where no reference group is defined) or Tukey’s test (for multiple comparisons where reference group is defined) as post-hoc analysis was used. If the variance across samples was tested to be significantly different, the Kruskal-Wallis test was used instead of ANOVA, with the Dunn test as the post-hoc multiple comparison test. Log-rank test was used for survival analysis. All statistical tests were performed in Graphpad software or R when statistical tests were not available through Graphpad.

## Supplementary Material

Supplementary Figures Fig. S1. Extended characterization of a CHIP-protective haplotype at the 17q22 locus.
**(A)** Global prevalence of the CHIP-protective haplotype across human populations. Figure generated by the Geography of Genetic Variants Browser (92). **(B)** Allele frequencies of CHIP-protective haplotype across different ancestries in the gnomAD database. **(C)** Meta-analysis of CHIP odds at the 17q22 CHIP-resilience haplotype shows a consistently protective effect across population biobanks examined across multiple studies (11, 26). AoU = All of Us; UKB = UK Biobank; GHS = Geisinger Health Study; MGB = Mass General Brigham; BioVU = Vanderbilt University Biobank. **(D)** LocusZoom plot shows high linkage disequilibrium (LD) between rs80093687 (11) and rs17834098 (26) reported in independent GWAS of CHIP-carriers), revealing a shared protective haplotype at the 17q22 locus. **(E)** Phenome-wide associations for CHIP-protective haplotype in UKB (11).
Fig. S2. Modeling CHIP-resilience variant effects in primary human HSPCs.
**(A)** ATAC-seq tracks at rs17834140 in primary hematopoietic cells and myeloid cell lines, demonstrating selective chromatin accessibility in human hematopoietic stem and multipotent progenitor cells. **(B)** Normalized RNA expression of genes within 1 Mb of rs17834140 in molecularly defined HSCs from human bone marrow. **(C)** Correlation between normalized ATAC-seq reads at rs17834140 and *MSI2* expression across hematopoietic cells. **(D)** Schematic of reporter assay conducted in primary human HSPCs to validate enhancer activity and assess variant effect. **(E)** Chromatin immunoprecipitation (ChIP)-qPCR showing high GATA2 occupancy at the *MSI2* enhancer in primary cord blood and adult CD34^+^ cells. **(F)** Representative flow cytometry plots showing strategy to either gate or sort CD34^+^CD45RA^−^CD90^+^ HSC-enriched cells for downstream analyses. **(G)** DNA editing efficiencies, inferred through Sanger ICE analysis or next-generation sequencing (NGS), 3 days after editing. **(H)** Editing outcomes at the *MSI2* enhancer by NGS, showing ~80% efficiency of ~100 bp microdeletion containing rs17834140. **(I)** Editing outcomes at MSI2 exon-targeting knockout (KO) by Sanger sequencing. **(J)** Relative *MSI2* expression 3 days following ENH-1 microdeletion by single-cell RNA-sequencing (scRNA-seq), within molecularly defined HSPC cell types. **(K)** Expression of genes within 1 Mb of rs17834140 in molecularly defined HSCs 3 days after editing at AAVS1 or ENH-1 microdeletion, by scRNA-seq.
Fig. S3. Extended characterization of the *MSI2* enhancer as a regulator of human HSCs.
**(A)** Growth curve showing overall fold expansion of adult CD34^+^ human hematopoietic stem and progenitor cells (HSPCs) following editing and culture in serum-free media. **(B)** Growth curve showing overall fold expansion of cord blood CD34^+^ human HSPCs following editing and culture in cytokine-free media. **(C)** Representative flow cytometry gating strategy used to assess human cell engraftment and lineage composition in bone marrow (BM) and spleen of mice 16 weeks after xenotransplantation. **(D)** Proportions of human CD45^+^ cells expressing lineage markers CD34 (HSPCs), CD19 (B-cells) or CD33 (myeloid cells), in BM or spleen of mice 16 weeks after xenotransplantation. **(E)** Representative colony forming assays (CFUs) in BM selected human CD34^+^ HSPCs plated at equal numbers and cultured in methylcellulose media for 14 days. (**F**) Quantification of CFUs showing increased differentiation capacity of ENH-1 and MSI2-KO edited cells.
Fig. S4. MSI2-HyperTRIBE and single cell profiling of human HSCs.
**(A)** Number of A>G edit sites detected in human cord blood CD34^+^ cells transduced with either empty vector or MSI2-ADAR (Adenosine Deaminases Acting on RNA enzyme). **(B)** Distribution of editing frequencies at 12,928 significant edit sites in MSI2-HyperTRIBE, defined by ≥10% difference in editing frequency between MSI2-ADAR and control samples, editing frequency in control samples <2%, and p<0.05. **(C)** Histogram showing the distribution of HyperTRIBE scores (sum of all differential editing frequencies per gene). **(D)** Venn diagram showing concordance of CD34^+^ MSI2-HyperTRIBE targets with MSI2-bound mRNAs by CLIP-seq (Cross-Linking Immunoprecipitation Sequencing) in NB4 hematopoietic cell line (33). **(E)** Genome tracks showing concordance of HyperTRIBE editing sites and CLIP-seq reads within 3’ UTR of mRNAs with focal (*TSPAN3*) or global (*CDK6*) MSI2 binding. **(F)** Uniform Manifold Approximation and Projection (UMAP) of all CD34^+^CD45RA^−^CD90^+^ HSPCs profiled by single-cell RNA sequencing (scRNA-seq), 72 hrs after editing at AAVS1, ENH-1 or MSI2-knockout. **(G)** UMAPs highlighting molecularly defined human HSCs from a human bone marrow reference map (85), which are enriched for expression of canonical HSC marker genes. **(H)** Scatter plot showing high correlation between log_2_FC of differentially expressed genes in ENH-1 vs. AAVS1 and MSI2-KO vs. AAVS1, supporting that microdeletion of the *MSI2* enhancer recapitulate effects of *MSI2* loss-of-function edits. **(I)** Inter-individual variation and positive correlation between *MSI2* and MSI2-DOWN CHIP-resilience network in HSCs among *n*=131 donors (39). **(J)** *ASXL1* mutant HSPCs are positively enriched for the MSI2-DOWN CHIP-resilience network (relative to *ASXL1* non-mutant HSPCs), in bone marrow CD34^+^ cells of patients with chronic myelomonocytic leukemia (CMML) (48). **(K)** CHIP-resilient (ENH-1 vs. AAVS1-edited) HSCs are negatively enriched for functional dependencies of *Dnmt3a*^R882H/+^ HSPCs (49). **(L)** Elevated *MSI2* expression at diagnosis predicts a poorer prognosis in pediatric AML (50). **(M)** Increased *MSI2* expression at diagnosis is associated with a more primitive adult AML subtype (51).
Fig. S5. Modeling germline-somatic interactions between rs17834140 and *ASXL1*-CHIP in human HSCs.
**(A)** Editing outcomes in primary human HSPCs edited with *ASXL1* exon 12 targeting single guide RNA (sgRNA), 7 days post-editing. **(B)** CRISPR model of *ASXL1* exon 12 targeting sgRNA expands phenotypic long-term (LT)-HSC maintenance in culture, recapitulating increased HSC fitness from patient mutations. **(C)** Editing efficiencies at two loci in primary HSPCs dually editing for somatic *ASXL1* mutation on a background of the germline resilience effect (AAVS1, ENH-1 or MSI2-KO), demonstrating that >75% of alleles are dually edited at both loci in culture. **(D)** Gene set enrichment analysis (GSEA) from RNA-seq of CD34^+^CD45RA^−^CD90^+^ HSPCs, showing that MSI2 loss (ENH-1 microdeletion or MSI2-knockout) reverses the transcriptional signature of *ASXL1*-mutant HSPCs. **(E)** *ASXL1* mutation induces an activated HSC signature in CD34^+^CD45RA^−^CD90^+^ HSPCs, which is attenuated under background of ENH-1 or MSI2-KO editing. **(F)** Representative secondary colonies formed by ±ENH-1 ±ASXL1 edited human CD34^+^ HSPCs. **(G)** Proportions of human CD45^+^ engrafted cells expressing CD117/KIT in bone marrow of NBSGW mice after xenotransplantation.
Fig. S6. *Asxl1* mutation and *MSI2* overexpression cooperatively induce MDS in mouse model.
**(A)** Flow cytometry quantifying MSI2 protein abundance in mouse bone marrow CD45^+^ cells, following *MSI2* induction by 1 month of doxycycline treatment. **(B)** Extended panel of peripheral blood traits in *Asxl1*^Δ/Δ^+*MSI2*^DOX^ murine model. RBC = red blood cell; Hb = hemoglobin; HCT = hematocrit; MCV = mean corpuscular volume; MCH = mean corpuscular hemoglobin; MPV = mean platelet volume. **(C)** Extended panel of peripheral blood (PB) and bone marrow (BM) morphology demonstrating myelodysplastic syndrome (MDS)-like features from *Asxl1*^Δ/Δ^+*MSI2*^DOX^ mice. All scale bars represent 20 μm.

Supplementary Tables Table S1: GWAS meta-analysis for CHIP.
Table S2: 17q22 CHIP-protective haplotype associations by CHIP driver mutations.
Table S3: Genetic and functional fine-mapping at CHIP-protective 17q22 haplotype.
**Table S4: CD34+ MSI2-HyperTRIBE binding targets.** Edit sites are aligned to human T2T CHM13-v1.1 reference genome.
Table S5: Differentially expressed genes for CHIP-resilience variant effect in human HSCs.
Table S6: MSI2-DOWN CHIP-resilience network. Table S7: Oligo and guide RNA sequences.

The PDF file includes:

Materials and Methods

Figs. S1 to S6

References (68-92)

Other Supplementary Material for this manuscript includes the following:

Tables S1 to S7
