# Supplementary material for: Inherited resilience to clonal hematopoiesis by modifying stem cell RNA regulation
Source: Science. Author manuscript; Available in PMC 2026 Jan 29. (PMC12850507; doi:10.1126/science.adx4174)
Supplement: Supplementary Figures — Fig. S1. Extended characterization of a CHIP-protective haplotype at the 17q22 locus. (A) Global prevalence of the CHIP-protective haplotype across human populations. Figure generated by the Geography of Genetic Variants Browser (92). (B) Allele frequencies of CHIP-protective haplotype across different ancestries in the gnomAD database. (C) Meta-analysis of CHIP odds at the 17q22 CHIP-resilience haplotype shows a consistently protective effect across population biobanks examined across multiple studies (11, 26). AoU = All of Us; UKB = UK Biobank; GHS = Geisinger Health Study; MGB = Mass General Brigham; BioVU = Vanderbilt University Biobank. (D) LocusZoom plot shows high linkage disequilibrium (LD) between rs80093687 (11) and rs17834098 (26) reported in independent GWAS of CHIP-carriers), revealing a shared protective haplotype at the 17q22 locus. (E) Phenome-wide associations for CHIP-protective haplotype in UKB (11). Fig. S2. Modeling CHIP-resilience variant effects in primary human HSPCs. (A) ATAC-seq tracks at rs17834140 in primary hematopoietic cells and myeloid cell lines, demonstrating selective chromatin accessibility in human hematopoietic stem and multipotent progenitor cells. (B) Normalized RNA expression of genes within 1 Mb of rs17834140 in molecularly defined HSCs from human bone marrow. (C) Correlation between normalized ATAC-seq reads at rs17834140 and MSI2 expression across hematopoietic cells. (D) Schematic of reporter assay conducted in primary human HSPCs to validate enhancer activity and assess variant effect. (E) Chromatin immunoprecipitation (ChIP)-qPCR showing high GATA2 occupancy at the MSI2 enhancer in primary cord blood and adult CD34+ cells. (F) Representative flow cytometry plots showing strategy to either gate or sort CD34+CD45RA−CD90+ HSC-enriched cells for downstream analyses. (G) DNA editing efficiencies, inferred through Sanger ICE analysis or next-generation sequencing (NGS), 3 days after editing. (H) Editing outcomes at the MSI2 e [file NIHMS2134378-supplement-Supplementary_Figures.pdf]

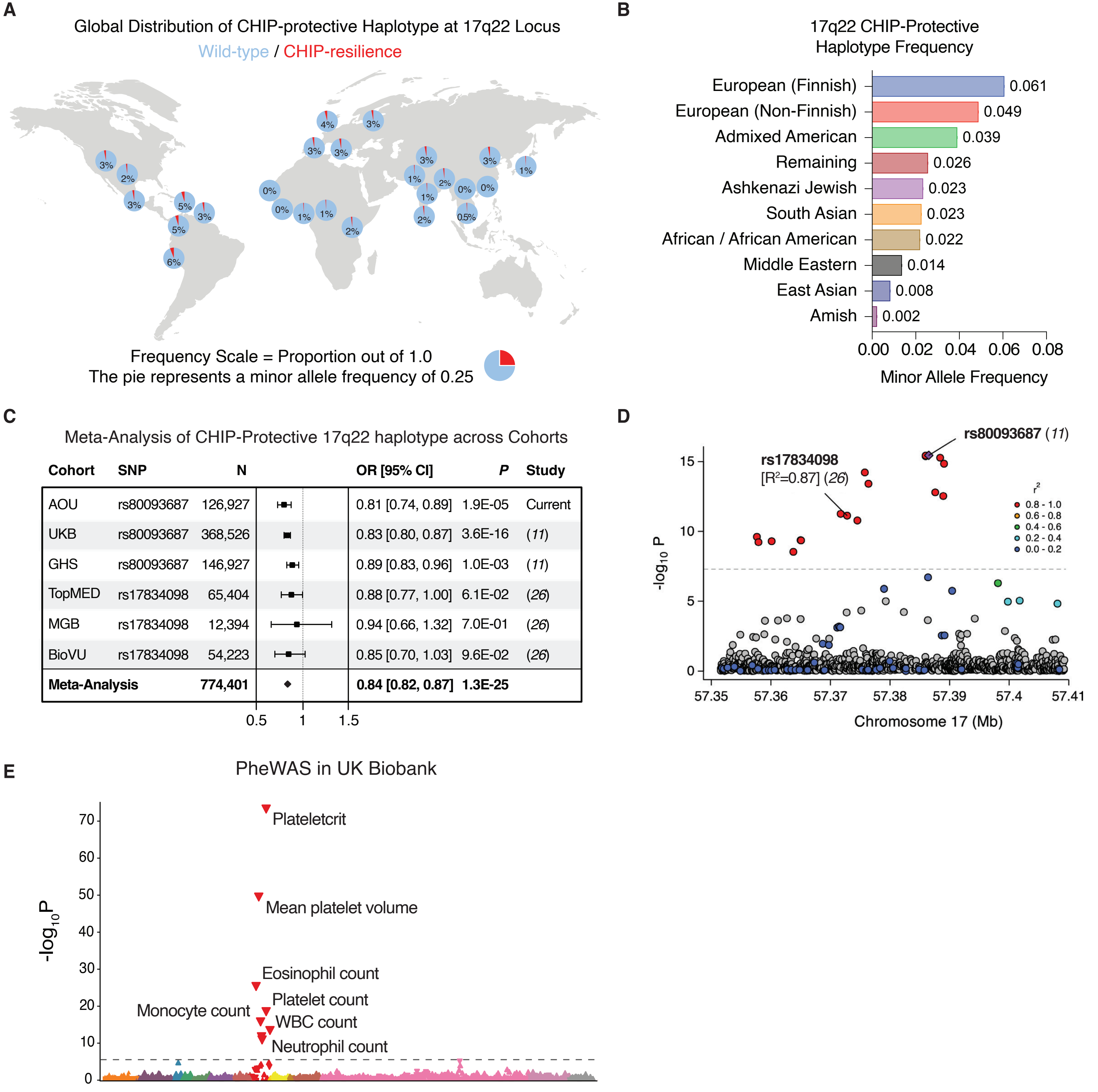

A
Global Distribution of CHIP-protective Haplotype at 17q22 Locus
Wild-type / CHIP-resilience
3%
2%
3%
5%
3%
5%
6%
0%
0%
1%
1%
2%
4%
3%
3%
3%
3%
1%
2%
1%
2%
0%
0%
0.5%
3%
1%
Frequency Scale = Proportion out of 1.0
The pie represents a minor allele frequency of 0.25
B
17q22 CHIP-Protective
Haplotype Frequency
European (Finnish) 0.061
European (Non-Finnish) 0.049
Admixed American 0.039
Remaining 0.026
Ashkenazi Jewish 0.023
South Asian 0.023
African / African American 0.022
Middle Eastern 0.014
East Asian 0.008
Amish 0.002
0.00 0.02 0.04 0.06 0.08
Minor Allele Frequency
C
Meta-Analysis of CHIP-Protective 17q22 haplotype across Cohorts
Cohort SNP N OR [95% CI] P Study
AOU rs80093687 126,927 0.81 [0.74, 0.89] 1.9E-05 Current
UKB rs80093687 368,526 0.83 [0.80, 0.87] 3.6E-16 (11)
GHS rs80093687 146,927 0.89 [0.83, 0.96] 1.0E-03 (11)
TopMED rs17834098 65,404 0.88 [0.77, 1.00] 6.1E-02 (26)
MGB rs17834098 12,394 0.94 [0.66, 1.32] 7.0E-01 (26)
BioVU rs17834098 54,223 0.85 [0.70, 1.03] 9.6E-02 (26)
Meta-Analysis 774,401 0.84 [0.82, 0.87] 1.3E-25
0.5 1 1.5
D
rs80093687 (11)
rs17834098
[R²=0.87] (26)
r²
0.8 - 1.0
0.6 - 0.8
0.4 - 0.6
0.2 - 0.4
0.0 - 0.2
-log10 P
15
10
5
0
57.35 57.36 57.37 57.38 57.39 57.4 57.41
Chromosome 17 (Mb)
E
PheWAS in UK Biobank
-log10P
70
60
50
40
30
20
10
0
Plateletcrit
Mean platelet volume
Eosinophil count
Platelet count
Monocyte count
WBC count
Neutrophil count

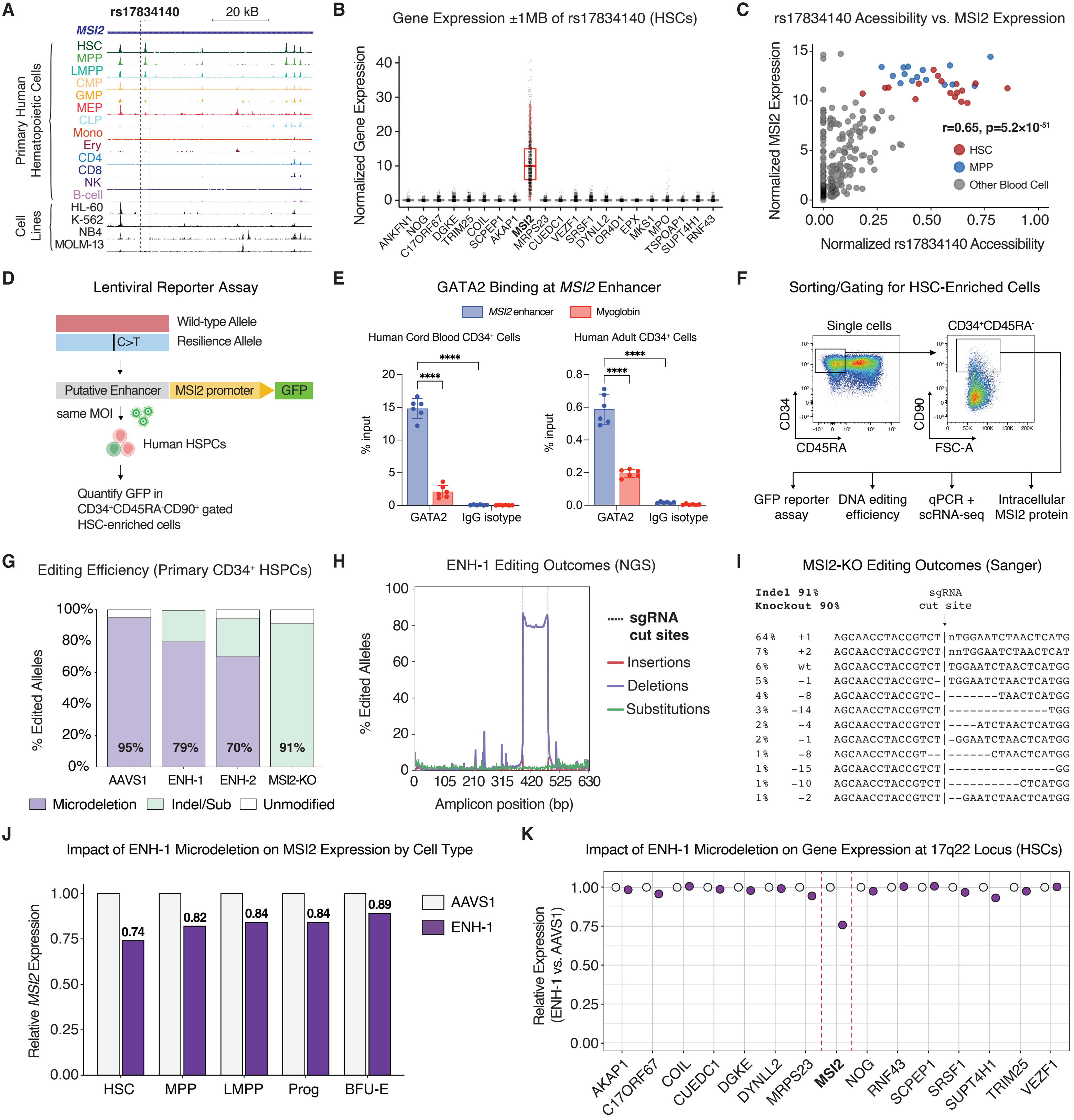
A
rs17834140
20 kB
MSI2
Primary Human Hematopoietic Cells
HSC
MPP
LMPP
CMP
GMP
MEP
CLP
Mono
Ery
CD4
CD8
NK
B-cell
Cell Lines
HL-60
K-562
NB4
MOLM-13
B
Gene Expression ±1MB of rs17834140 (HSCs)
Normalized Gene Expression
ANKFN1
NOG
C17ORF67
DGKE
TRIM25
COIL
SCPEP1
AKAP1
MSI2
MRPS23
CUEDC1
VEZF1
SRSF1
DYNLL2
OR4D1
EPX
MKS1
MPO
TSPOAP1
SUPT4H1
RNF43
C
rs17834140 Acessibility vs. MSI2 Expression
Normalized MSI2 Expression
Normalized rs17834140 Accessibility
r=0.65, p=5.2x10-51
HSC
MPP
Other Blood Cell
D
Lentiviral Reporter Assay
Wild-type Allele
C>T
Resilience Allele
Putative Enhancer
MSI2 promoter
GFP
same MOI
Human HSPCs
Quantify GFP in CD34+CD45RA-CD90+ gated HSC-enriched cells
E
GATA2 Binding at MSI2 Enhancer
MSI2 enhancer
Myoglobin
Human Cord Blood CD34+ Cells
Human Adult CD34+ Cells
% input
GATA2
IgG isotype
F
Sorting/Gating for HSC-Enriched Cells
Single cells
CD34+CD45RA-
CD34
CD45RA
CD90
FSC-A
GFP reporter assay
DNA editing efficiency
qPCR + scRNA-seq
Intracellular MSI2 protein
G
Editing Efficiency (Primary CD34+ HSPCs)
% Edited Alleles
95%
79%
70%
91%
AAVS1
ENH-1
ENH-2
MSI2-KO
Microdeletion
Indel/Sub
Unmodified
H
ENH-1 Editing Outcomes (NGS)
% Edited Alleles
Amplicon position (bp)
sgRNA cut sites
Insertions
Deletions
Substitutions
I
MSI2-KO Editing Outcomes (Sanger)
Indel 91%
Knockout 90%
sgRNA cut site
64% +1 AGCAACCTACCGTCT|nTGGAATCTAACTCATG
7% +2 AGCAACCTACCGTCT|nnTGGAATCTAACTCAT
6% wt AGCAACCTACCGTCT|TGGAATCTAACTCATGG
5% -1 AGCAACCTACCGTC-|TGGAATCTAACTCATGG
4% -8 AGCAACCTACCGTC-|-------TAACTCATGG
3% -14 AGCAACCTACCGTCT|--------------TGG
2% -4 AGCAACCTACCGTCT|----ATCTAACTCATGG
2% -1 AGCAACCTACCGTCT|-GGAATCTAACTCATGG
1% -8 AGCAACCTACCGT--|------CTAACTCATGG
1% -15 AGCAACCTACCGTCT|---------------GG
1% -10 AGCAACCTACCGTCT|----------CTCATGG
1% -2 AGCAACCTACCGTCT|--GAATCTAACTCATGG
J
Impact of ENH-1 Microdeletion on MSI2 Expression by Cell Type
Relative MSI2 Expression
0.74
0.82
0.84
0.84
0.89
HSC
MPP
LMPP
Prog
BFU-E
AAVS1
ENH-1
K
Impact of ENH-1 Microdeletion on Gene Expression at 17q22 Locus (HSCs)
Relative Expression (ENH-1 vs. AAVS1)
AKAP1
C17ORF67
COIL
CUEDC1
DGKE
DYNLL2
MRPS23
MSI2
NOG
RNF43
SCPEP1
SRSF1
SUPT4H1
TRIM25
VEZF1

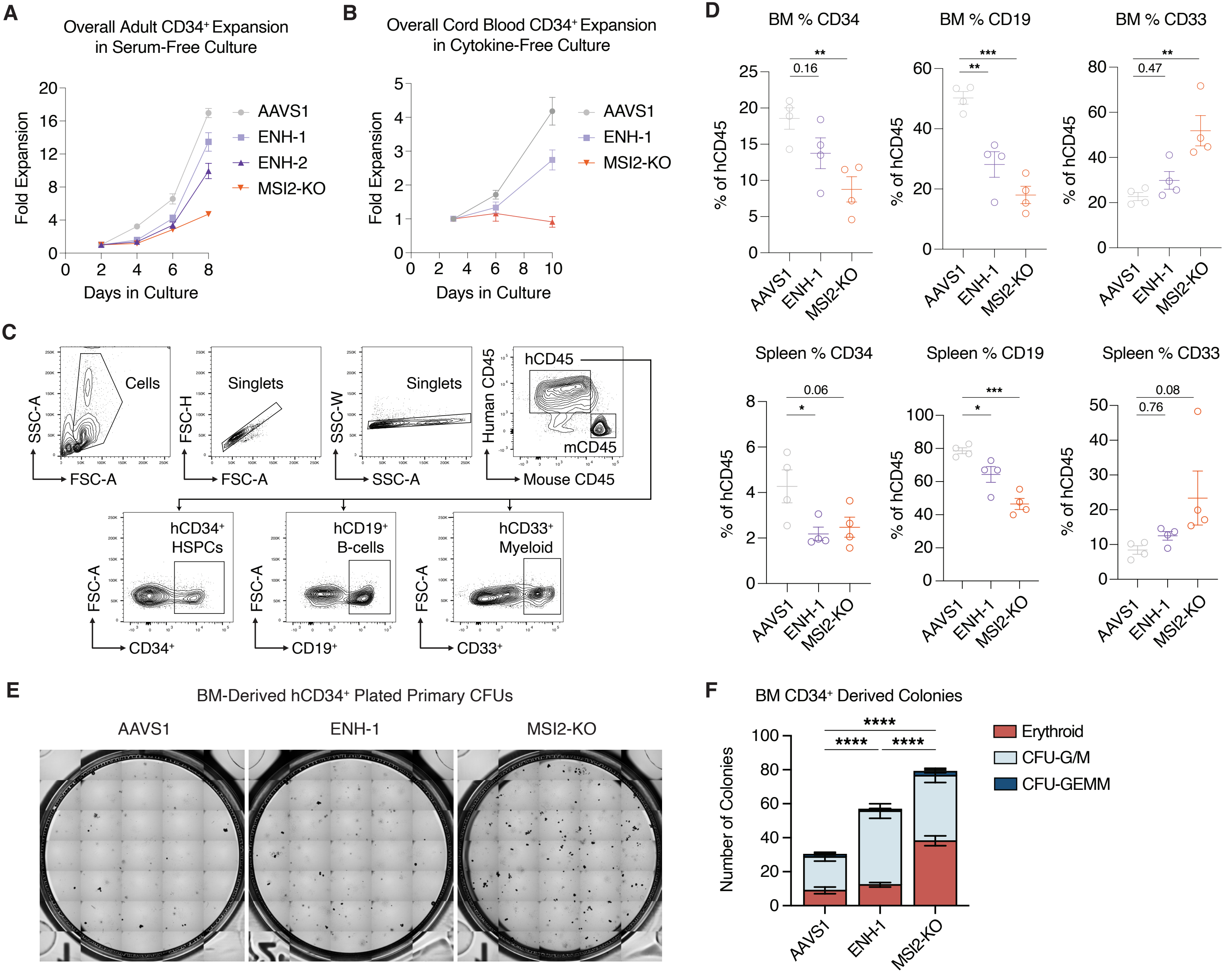
A
Overall Adult CD34+ Expansion in Serum-Free Culture
Fold Expansion
Days in Culture
AAVS1
ENH-1
ENH-2
MSI2-KO
B
Overall Cord Blood CD34+ Expansion in Cytokine-Free Culture
Fold Expansion
Days in Culture
AAVS1
ENH-1
MSI2-KO
C
Cells
Singlets
Singlets
hCD45
mCD45
SSC-A
FSC-A
FSC-H
FSC-A
SSC-W
SSC-A
Human CD45
Mouse CD45
hCD34+ HSPCs
hCD19+ B-cells
hCD33+ Myeloid
FSC-A
CD34+
CD19+
CD33+
D
BM % CD34
BM % CD19
BM % CD33
Spleen % CD34
Spleen % CD19
Spleen % CD33
% of hCD45
AAVS1
ENH-1
MSI2-KO
0.16
0.47
0.06
0.76
0.08
E
BM-Derived hCD34+ Plated Primary CFUs
AAVS1
ENH-1
MSI2-KO
F
BM CD34+ Derived Colonies
Number of Colonies
Erythroid
CFU-G/M
CFU-GEMM
AAVS1
ENH-1
MSI2-KO

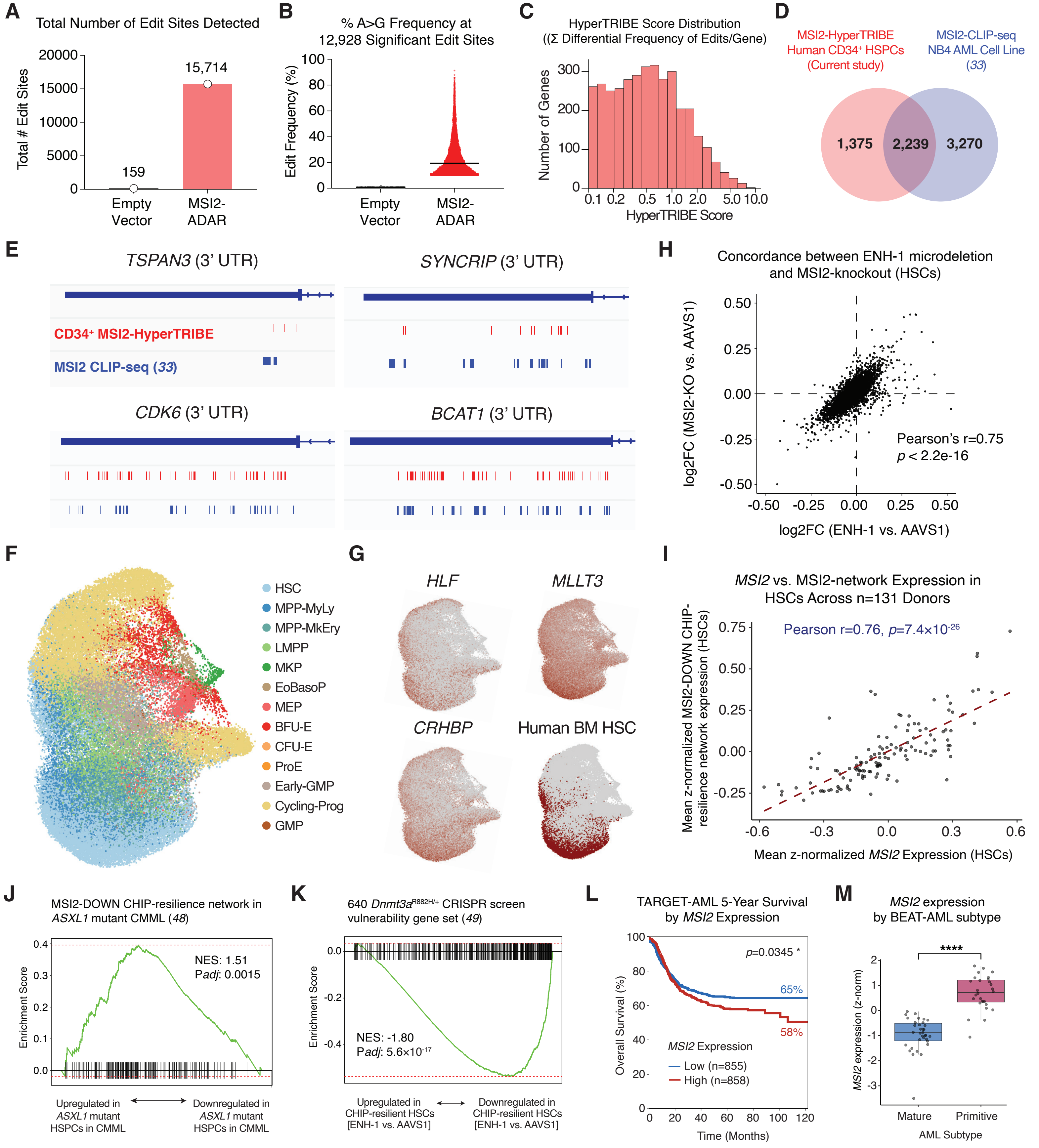
A
Total Number of Edit Sites Detected
Total # Edit Sites
15,714
159
Empty Vector
MSI2-ADAR
B
% A>G Frequency at 12,928 Significant Edit Sites
Edit Frequency (%)
Empty Vector
MSI2-ADAR
C
HyperTRIBE Score Distribution
((Σ Differential Frequency of Edits/Gene)
Number of Genes
HyperTRIBE Score
D
MSI2-HyperTRIBE Human CD34+ HSPCs (Current study)
MSI2-CLIP-seq NB4 AML Cell Line (33)
1,375
2,239
3,270
E
TSPAN3 (3' UTR)
SYNCRIP (3' UTR)
CD34+ MSI2-HyperTRIBE
MSI2 CLIP-seq (33)
CDK6 (3' UTR)
BCAT1 (3' UTR)
H
Concordance between ENH-1 microdeletion and MSI2-knockout (HSCs)
log2FC (MSI2-KO vs. AAVS1)
log2FC (ENH-1 vs. AAVS1)
Pearson's r=0.75
p < 2.2e-16
F
HSC
MPP-MyLy
MPP-MkEry
LMPP
MKP
EoBasoP
MEP
BFU-E
CFU-E
ProE
Early-GMP
Cycling-Prog
GMP
G
HLF
MLLT3
CRHBP
Human BM HSC
I
MSI2 vs. MSI2-network Expression in HSCs Across n=131 Donors
Pearson r=0.76, p=7.4×10-26
Mean z-normalized MSI2-DOWN CHIP-resilience network expression (HSCs)
Mean z-normalized MSI2 Expression (HSCs)
J
MSI2-DOWN CHIP-resilience network in ASXL1 mutant CMML (48)
Enrichment Score
NES: 1.51
Padj: 0.0015
Upregulated in ASXL1 mutant HSPCs in CMML
Downregulated in ASXL1 mutant HSPCs in CMML
K
640 Dnmt3aR882H/+ CRISPR screen vulnerability gene set (49)
Enrichment Score
NES: -1.80
Padj: 5.6×10-17
Upregulated in CHIP-resilient HSCs [ENH-1 vs. AAVS1]
Downregulated in CHIP-resilient HSCs [ENH-1 vs. AAVS1]
L
TARGET-AML 5-Year Survival by MSI2 Expression
Overall Survival (%)
p=0.0345 *
65%
58%
MSI2 Expression
Low (n=855)
High (n=858)
Time (Months)
M
MSI2 expression by BEAT-AML subtype
MSI2 expression (z-norm)
****
Mature
Primitive
AML Subtype

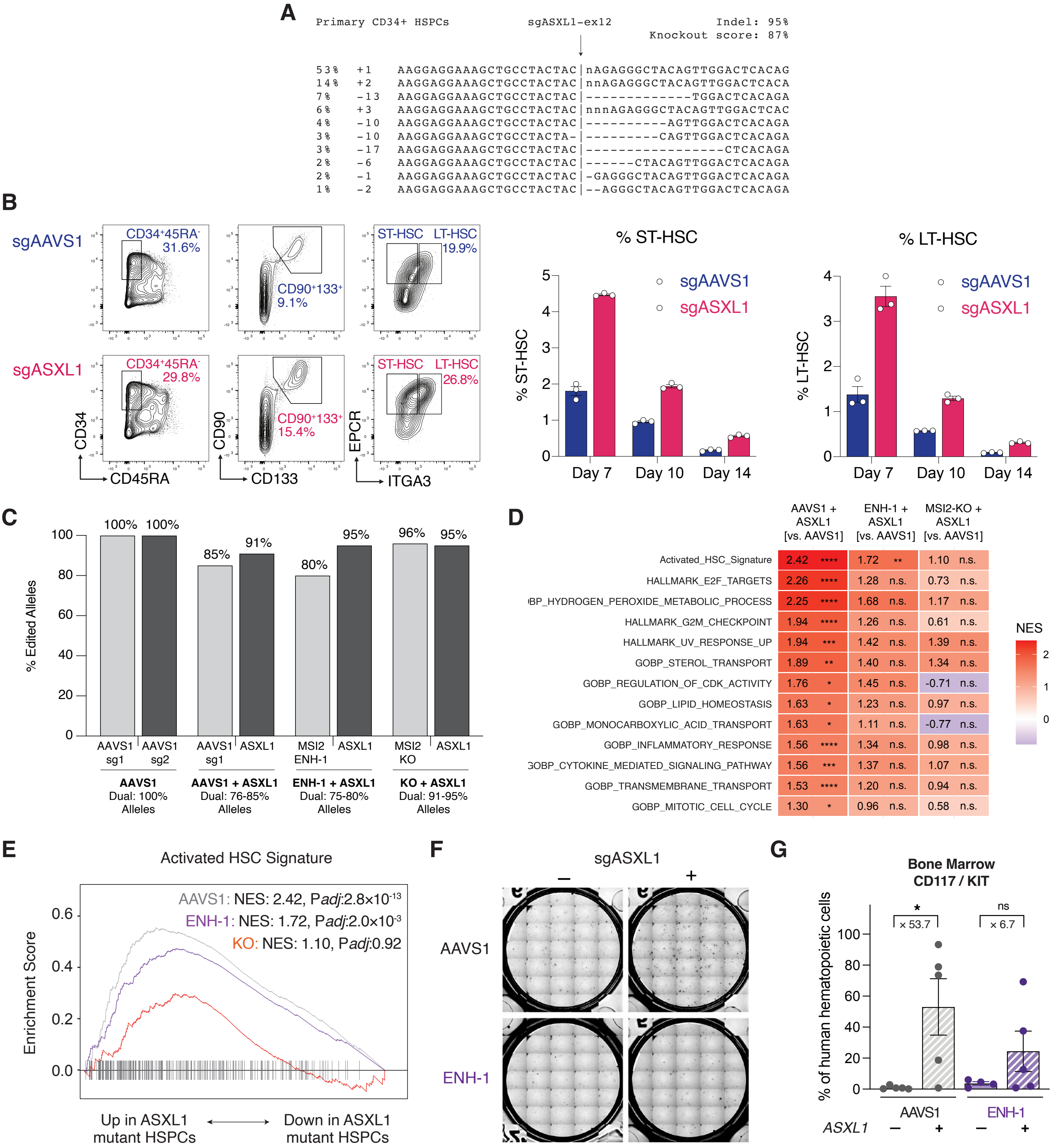
A
Primary CD34+ HSPCs
sgASXL1-ex12
Indel: 95%
Knockout score: 87%
53% +1 AAGGAGGAAAGCTGCCTACTAC|nAGAGGGCTACAGTTGGACTCACAG
14% +2 AAGGAGGAAAGCTGCCTACTAC|nnAGAGGGCTACAGTTGGACTCACA
7% -13 AAGGAGGAAAGCTGCCTACTAC|-------------TGGACTCACAGA
6% +3 AAGGAGGAAAGCTGCCTACTAC|nnnAGAGGGCTACAGTTGGACTCAC
4% -10 AAGGAGGAAAGCTGCCTACTAC|----------AGTTGGACTCACAGA
3% -10 AAGGAGGAAAGCTGCCTACTA-|---------CAGTTGGACTCACAGA
3% -17 AAGGAGGAAAGCTGCCTACTAC|-----------------CTCACAGA
2% -6 AAGGAGGAAAGCTGCCTACTAC|------CTACAGTTGGACTCACAGA
2% -1 AAGGAGGAAAGCTGCCTACTAC|-GAGGGCTACAGTTGGACTCACAGA
1% -2 AAGGAGGAAAGCTGCCTACTAC|--AGGGCTACAGTTGGACTCACAGA
B
sgAAVS1
CD34+45RA-
31.6%
CD90+133+
9.1%
ST-HSC
LT-HSC
19.9%
sgASXL1
CD34+45RA-
29.8%
CD90+133+
15.4%
ST-HSC
LT-HSC
26.8%
CD34
CD45RA
CD90
CD133
EPCR
ITGA3
% ST-HSC
% LT-HSC
sgAAVS1
sgASXL1
Day 7
Day 10
Day 14
C
% Edited Alleles
100% 100% 85% 91% 80% 95% 96% 95%
AAVS1 sg1
AAVS1 sg2
AAVS1 sg1
ASXL1
MSI2 ENH-1
ASXL1
MSI2 KO
ASXL1
AAVS1
Dual: 100% Alleles
AAVS1 + ASXL1
Dual: 76-85% Alleles
ENH-1 + ASXL1
Dual: 75-80% Alleles
KO + ASXL1
Dual: 91-95% Alleles
D
AAVS1 + ASXL1 [vs. AAVS1]
ENH-1 + ASXL1 [vs. AAVS1]
MSI2-KO + ASXL1 [vs. AAVS1]
Activated_HSC_Signature 2.42 **** 1.72 ** 1.10 n.s.
HALLMARK_E2F_TARGETS 2.26 **** 1.28 n.s. 0.73 n.s.
BP_HYDROGEN_PEROXIDE_METABOLIC_PROCESS 2.25 **** 1.68 n.s. 1.17 n.s.
HALLMARK_G2M_CHECKPOINT 1.94 **** 1.26 n.s. 0.61 n.s.
HALLMARK_UV_RESPONSE_UP 1.94 *** 1.42 n.s. 1.39 n.s.
GOBP_STEROL_TRANSPORT 1.89 ** 1.40 n.s. 1.34 n.s.
GOBP_REGULATION_OF_CDK_ACTIVITY 1.76 * 1.45 n.s. -0.71 n.s.
GOBP_LIPID_HOMEOSTASIS 1.63 * 1.23 n.s. 0.97 n.s.
GOBP_MONOCARBOXYLIC_ACID_TRANSPORT 1.63 * 1.11 n.s. -0.77 n.s.
GOBP_INFLAMMATORY_RESPONSE 1.56 **** 1.34 n.s. 0.98 n.s.
GOBP_CYTOKINE_MEDIATED_SIGNALING_PATHWAY 1.56 *** 1.37 n.s. 1.07 n.s.
GOBP_TRANSMEMBRANE_TRANSPORT 1.53 **** 1.20 n.s. 0.94 n.s.
GOBP_MITOTIC_CELL_CYCLE 1.30 * 0.96 n.s. 0.58 n.s.
NES
E
Activated HSC Signature
AAVS1: NES: 2.42, Padj:2.8×10-13
ENH-1: NES: 1.72, Padj:2.0×10-3
KO: NES: 1.10, Padj:0.92
Enrichment Score
Up in ASXL1 mutant HSPCs
Down in ASXL1 mutant HSPCs
F
sgASXL1
–
+
AAVS1
ENH-1
G
Bone Marrow CD117 / KIT
% of human hematopoietic cells
*
× 53.7
ns
× 6.7
AAVS1
ENH-1
ASXL1 – + – +

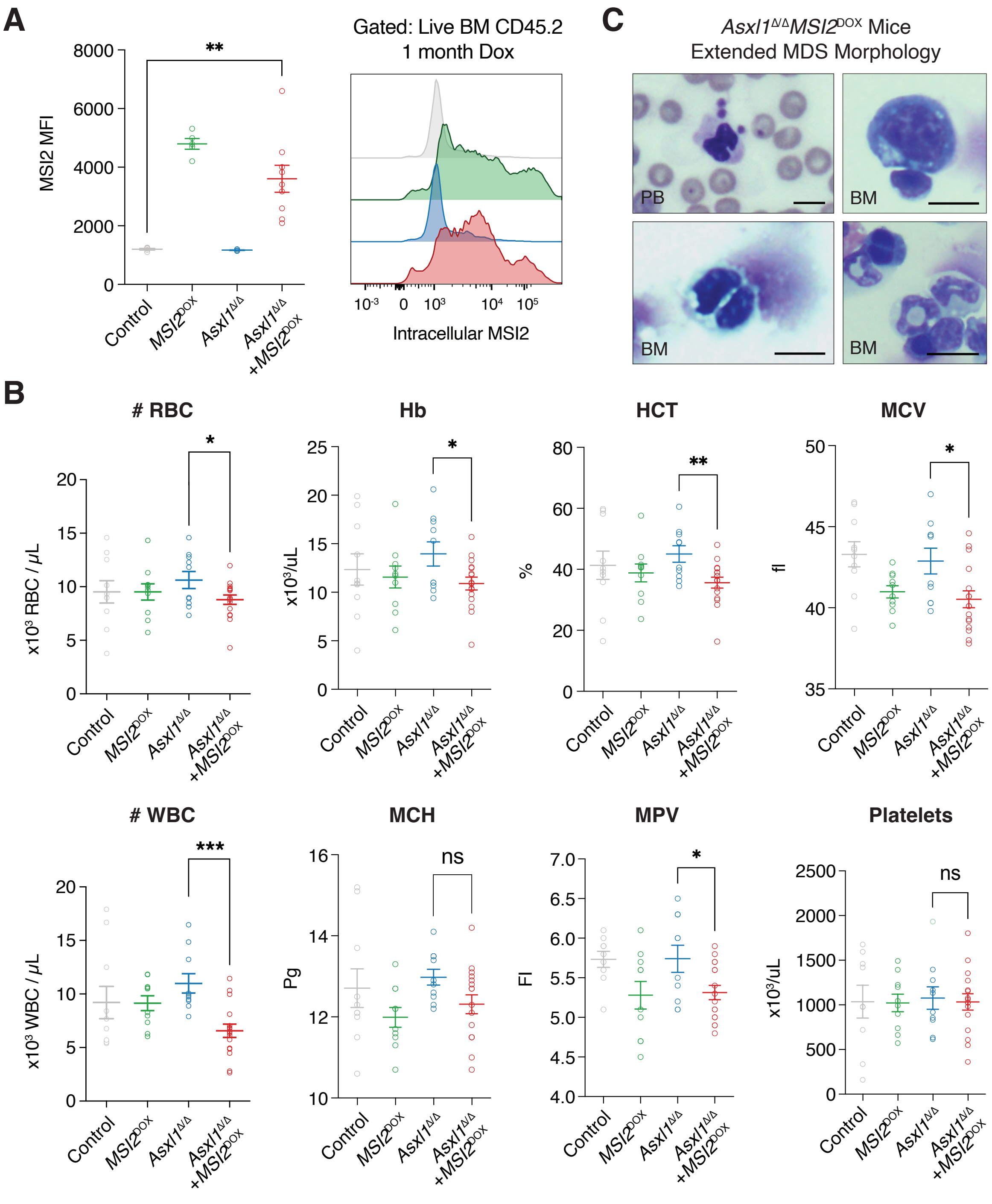

A
MSI2 MFI
8000
6000
4000
2000
0
**
Control
MSI2DOX
Asxl1Δ/Δ
Asxl1Δ/Δ +MSI2DOX
Gated: Live BM CD45.2
1 month Dox
Intracellular MSI2
C
Asxl1Δ/ΔMSI2DOX Mice
Extended MDS Morphology
PB
BM
BM
BM
B
RBC
x10³ RBC / μL
*
Hb
x10³/uL
*
HCT
%
**
MCV
fl
*
WBC
x10³ WBC / μL
***
MCH
Pg
ns
MPV
Fl
*
Platelets
x10³/uL
ns
